# Seroconversion Rates Following COVID-19 Vaccination in People Living with HIV: A Systematic Review and Meta-Analysis

**DOI:** 10.3390/vaccines14080648

**Published:** 2026-07-23

**Authors:** Maya Alkhidir, Kannan Sridharan

**Affiliations:** 1 College of Medicine & Health Sciences, Arabian Gulf University, Manama 329, Bahrain; mayakhidir04@gmail.com; 2 Department of Pharmacology & Therapeutics, College of Medicine & Health Sciences, Arabian Gulf University, Manama 329, Bahrain

**Keywords:** PLWH, COVID-19, seroconversion, immunosuppressed

## Abstract

**Background**: People living with human immunodeficiency virus (HIV) (PLWH) remain at increased risk of severe COVID-19 outcomes; however, conflicting evidence exists regarding the seroconversion rates of COVID-19 vaccines in this population. **Methods**: A systematic review and meta-analysis were conducted on studies reporting seroconversion outcomes following COVID-19 vaccination in PLWH. **Results**: Forty-four studies (5391 PLWH) were included in the meta-analysis. The overall pooled seroconversion proportion was 93.5%. Bootstrap analysis confirmed robustness (92.8%). Subgroup analyses revealed significantly higher seroconversion rates for mRNA vaccines (98.2%) compared to non-mRNA vaccines (80.5%). CD4 count demonstrated a graded association: <200 cells/mm^3^ (53.8%), 200–500 cells/mm^3^ (86.2%), and >500 cells/mm^3^ (93.5%). Prior COVID-19 infection (99.1% vs. 89.5%) and antiretroviral therapy (ART) status (93.5% vs. 49.6%) were significant determinants. Safety data demonstrated a favorable profile, with predominantly mild-to-moderate local (injection-site pain: 23.8%) and systemic (headache: 12.95%, fatigue: 6.4%) adverse events; serious adverse events were rare and no consistent association with HIV disease progression was observed. **Conclusions**: COVID-19 vaccination induces high seroconversion rates in PLWH, particularly among those receiving mRNA vaccines, with preserved CD4 counts, on ART, or with prior infection.

## 1. Introduction

The declaration of COVID-19 as a global pandemic on 11 March 2020, prompted a coordinated international response focused on the rapid development of a vaccine to address elevated morbidity and mortality rates. Numerous organizations, governmental bodies, academic institutions, and research centres collaborated to accelerate vaccine development, which resulted in the rapid deployment of multiple vaccine platforms across the globe [1,2]. Since their introduction, vaccines have been the cornerstone of public health prevention strategies, significantly reducing hospitalizations, deaths, and severe disease.

Although COVID-19 vaccines have achieved remarkable success in the general population, several concerns remain about their efficacy and safety in immunocompromised patients, whose impaired immune systems may limit their ability to mount adequate protective antibody responses. Immunocompromised patients are individuals with a deficient immune response due to weakened immune mechanisms resulting from an underlying disease or disorder such as those infected with human immunodeficiency virus (HIV), or from the administration of drugs or radiation therapy [3].

Among the immunocompromised patients, people living with HIV (PLWH) represent a particularly significant group. HIV belongs to the Retroviridae family, which is in the Lentivirus genus. This virus targets CD4+ T helper cells, leading to severe immune suppression and ongoing cell loss. Immune system suppression weakens the immune system, leading to various clinical manifestations. Untreated HIV would clinically progress to AIDs. At this stage, the immune system is unable to prevent opportunistic infections, which can be fatal. HIV is a global public health challenge. In accordance with the WHO HIV factsheet, there were 40.8 million people living with HIV (PLWH) at the end of 2024, of whom more than 60% living in Sub-Saharan Africa [4].

Additionally, the immune dysfunction associated with HIV increases the patient’s vulnerability to COVID-19. Patients who exhibit an uncontrolled HIV infection, low CD4 count, and unsuppressed viral load are at a particularly high risk of severe COVID-19, hospitalizations, or mortality. This risk is further amplified by the high number of PLWH patients suffering from various comorbidities, such as obesity, hypertension, and diabetes. This is evidenced by the recent studies have demonstrated an increased risk of severe COVID-19 in PLWH, and numerous meta-analyses have simplified these findings, confirming that HIV infection is associated with a higher risk of death from COVID-19. This underscores the importance of effective vaccination strategies to safeguard this population’s safety [5]. Hence, recognizing these compounded risks makes understanding the immunologic response to vaccines in HIV-positive individuals highly crucial [5].

Against this background, vaccination against COVID-19 remains one of the most effective ways to prevent its most serious complications worldwide, for immunocompetent and immunocompromised patients. It has been proven that vaccination reduces COVID-19-related serious outcomes, like mortality and severe illness, and this is particularly significant for patients who have a weak immune system, like PLWH [5]. Multiple vaccine platforms have been developed, including mRNA (BNT162b2, mRNA-1273), adenoviral vector (ChAdOx1 nCoV-19, Ad26.COV2.S), and inactivated virus (BBIBP-CorV, CoronaVac). All these vaccines were widely deployed during the pandemic [6]. Primary vaccination studies have demonstrated that PLWH, across different ethnic and geographic backgrounds, tend to develop protective antibodies and exhibit a reduced risk of COVID-19 post-vaccination. However, several studies have shown that the serological response remains lower in PLWH than in healthy individuals [7].

As previously discussed, HIV-associated immunosuppression could influence the progression of COVID-19 symptoms, emphasizing the need for close monitoring of the PLWH population. Equally important is assessing the effectiveness and safety of the various COVID-19 vaccines administered to these patients. Conflicting evidence exists regarding the safety and immunogenicity of different vaccine platforms in PLWH: some studies report attenuated immunogenicity and efficacy of COVID-19 vaccines, while others report results comparable to healthy controls. Safety analyses similarly vary, with some studies finding increased adverse events after vaccination in PLWH compared with healthy controls, while others do not. Hence, a holistic understanding of the issue is essential [6,7,8].

Although there is a rapid expansion of the literature examining the COVID-19 vaccine response in people living with HIV, a consistent and clear understanding of the immunogenicity and safety across the differing vaccine platforms has not been analyzed or presented in detail and remains lacking. Existing studies (including in healthy adults) and meta-analyses report conflicting findings: some demonstrate significantly reduced seroconversion rates and antibody titers in PLWH, while others suggest that immune responses to the vaccine in PLWH are equivalent to those in immunocompetent people [5,6,7,8,9,10,11,12,13,14]. Additionally, the current meta-analysis hasn’t comprehensively stratified outcomes by key clinical variables, such as the degree of immunosuppression, comorbidity burden, and vaccine platform, which are critical determinants of vaccine efficacy in this population. Inconsistencies in safety profiles often contribute to uncertainty in clinical decision-making. We hypothesized that COVID-19 vaccination would induce robust seroconversion rates in PLWH, with mRNA vaccines proving superior to non-mRNA platforms, and that responses would be positively associated with higher CD4+ T-cell counts, ART use, and prior SARS-CoV-2 infection, while maintaining a favorable safety profile without impacting HIV disease progression. Hence, this study aims to conduct a systematic review and meta-analysis to address an important research gap by providing a unified, quantitative, and robust synthesis of the immunogenicity and safety outcomes of COVID-19 vaccines in PLWH, along with appropriate subgroup analyses, to clarify the influence of immunological status and vaccine type. 

## 2. Methods

### 2.1. Search Methods

The protocol for this ancillary systematic review was prospectively registered in the Open Science Framework and was also the basis for a previous systematic review [15,16]. A comprehensive literature search was conducted across three electronic databases: Medline (via PubMed), Cochrane Central Register of Controlled Trials (CENTRAL), and Google Scholar, to identify eligible studies that evaluated the efficacy and safety of COVID-19 vaccines in PLWH. Details of the search strategy are summarized in the Electronic Supplementary Table S1. The search was last updated on 15 May 2026. We did not place any restrictions regarding the language or year of the publications. We excluded conference abstracts, reviews and editorials. References from the eligible articles were examined and references pertinent to this study were included.

### 2.2. Eligibility Criteria

Population: The included studies involved immunocompromised patients, defined as individuals with impaired immune function due to underlying conditions or treatment. This study included and focused on PLWH.

**Intervention**: The intervention of interest was the administration of any WHO- or FDA-authorized COVID-19 vaccine, including mRNA vaccines such as BNT162b2 (Pfizer–BioNTech) and mRNA-1273 (Moderna), and Viral vector vaccines such as AZD1222 (Oxford–AstraZeneca) and Ad26.COV2.S (Janssen/Johnson & Johnson), Protein subunit vaccines: NVX-CoV2373 (Novavax), Inactivated vaccines: BBIBP-CorV (Sinopharm), CoronaVac (Sinovac), where applicable. Studies assessing both primary vaccination series and booster (additional) doses were included.

**Comparator:** Immunocompetent vaccinated individuals, Unvaccinated immunocompromised individuals, Immunocompromised patients receiving different immunosuppressive regimens or with varying degrees of immune suppression, Placebo groups or historical controls, where available.

### 2.3. Outcomes

- Seroconversion rate: defined as the proportion of participants achieving antibody levels above a predefined assay-specific threshold. We did not adhere to any specific definitions but carried out additional sub-group analyses based on the definitions used by individual authors.
- Safety profile: Local adverse events (e.g., injection-site pain, erythema), Systemic adverse events (e.g., fever, fatigue, myalgia), Serious adverse events (SAEs), including hospitalization, disease exacerbation, or other clinically significant outcomes related to vaccination.

Study Design: We included randomized controlled trials, cohort studies, and observational studies reporting relevant seroconversion or safety outcomes. Case reports, reviews, editorials, and studies lacking sufficient data were excluded.

### 2.4. Statistical Analysis Methods

#### 2.4.1. Data Preparation and Transformation

Prior to analysis, studies with multiple entries due to different vaccine types or dosing regimens were aggregated by summing the total participants and seroconverted events for each unique study identifier to avoid unit-of-analysis errors. For subgroup analyses, the original disaggregated data were retained to allow for within-study comparisons across different vaccine characteristics. The Freeman-Tukey double arcsine transformation was applied to stabilize variances and normalize the distribution of proportions before pooling, as this transformation is particularly suitable for meta-analyses of proportions where event rates may approach the boundaries of zero or one. All pooled estimates were back-transformed to the original proportion scale using the inverse Freeman-Tukey transformation.

#### 2.4.2. Overall Pooled Proportion and Heterogeneity Assessment

A random-effects meta-analysis model using restricted maximum likelihood estimation was employed to calculate the overall pooled seroconversion proportion across all included studies. The Hartung–Knapp–Sidik–Jonkman adjustment was applied to provide more robust confidence intervals that account for uncertainty in the estimation of between-study variance, which is particularly important when the number of studies is small or heterogeneity is substantial. Heterogeneity was assessed using the Cochrane Q statistic and quantified using the I^2^ statistic, which represents the percentage of total variation across studies that is attributable to heterogeneity rather than chance. The between-study variance was estimated using the restricted maximum likelihood method, and a 95% prediction interval was calculated to estimate the range within which the true effect of a future study would fall, providing a measure of the clinical applicability of the findings across different settings.

#### 2.4.3. Internal Validation Through Bootstrap Analysis

A bootstrap resampling procedure with 1000 iterations was performed to internally validate the robustness of the pooled estimate. For each iteration, a random sample of studies with replacement was drawn, the meta-analysis was re-estimated, and the distribution of the pooled estimates across all iterations was used to construct empirical confidence intervals. A fixed random seed (123) was set to ensure the reproducibility of the bootstrap results.

#### 2.4.4. Trial Sequential Analysis

A trial sequential analysis was conducted to evaluate whether the cumulative evidence was sufficient to reach a firm conclusion and to control the risk of random errors due to sparse data or repeated significance testing. The required information size was calculated based on the observed event proportion, with alpha set at 0.05 for type I error and beta at 0.20 for type II error, corresponding to 80% statistical power. The O’Brien-Fleming alpha spending function was applied to construct monitoring boundaries, and the cumulative Z-statistic was plotted against the information fraction to determine whether the evidence crossed the trial sequential monitoring boundaries or the futility boundaries.

#### 2.4.5. Sensitivity and Publication Bias Analyses

A leave-one-out sensitivity analysis was conducted to evaluate the influence of individual studies on the overall pooled estimate, whereby each study was systematically removed and the meta-analysis re-run to assess the stability of the findings. Studies identified as potentially influential were examined to determine whether their exclusion materially altered the conclusions. Publication bias was systematically evaluated using Egger’s regression test to assess asymmetry by regressing the standardized effect estimates against their precision. Risk of bias was assessed using Cochrane risk of bias tool for randomized trials and JBI checklist for observational studies.

#### 2.4.6. Exploratory Subgroup Analyses

Predefined subgroup analyses were conducted to explore potential sources of heterogeneity and to examine whether the pooled seroconversion proportion varied according to clinically relevant study-level characteristics. Subgroup analyses were performed for assay type, prior COVID-19 infection status, seroconversion definition criteria, mRNA versus non-mRNA vaccine type, CD4 count categories, study design, specific vaccines administered, number of vaccine doses, and antiretroviral therapy status. Only subgroups with a minimum of two studies were included in the analysis to ensure reliable estimation. For each subgroup, the pooled proportion with 95% confidence interval was calculated using the same random-effects model with REML estimation and HKSJ adjustment.

All statistical tests were two-sided, and a *p*-value ≤ 0.05 was considered statistically significant. All data processing and statistical analyses were carried out in R (version 4.5.2).

## 3. Results

### 3.1. Search Results

**Figure 1:**
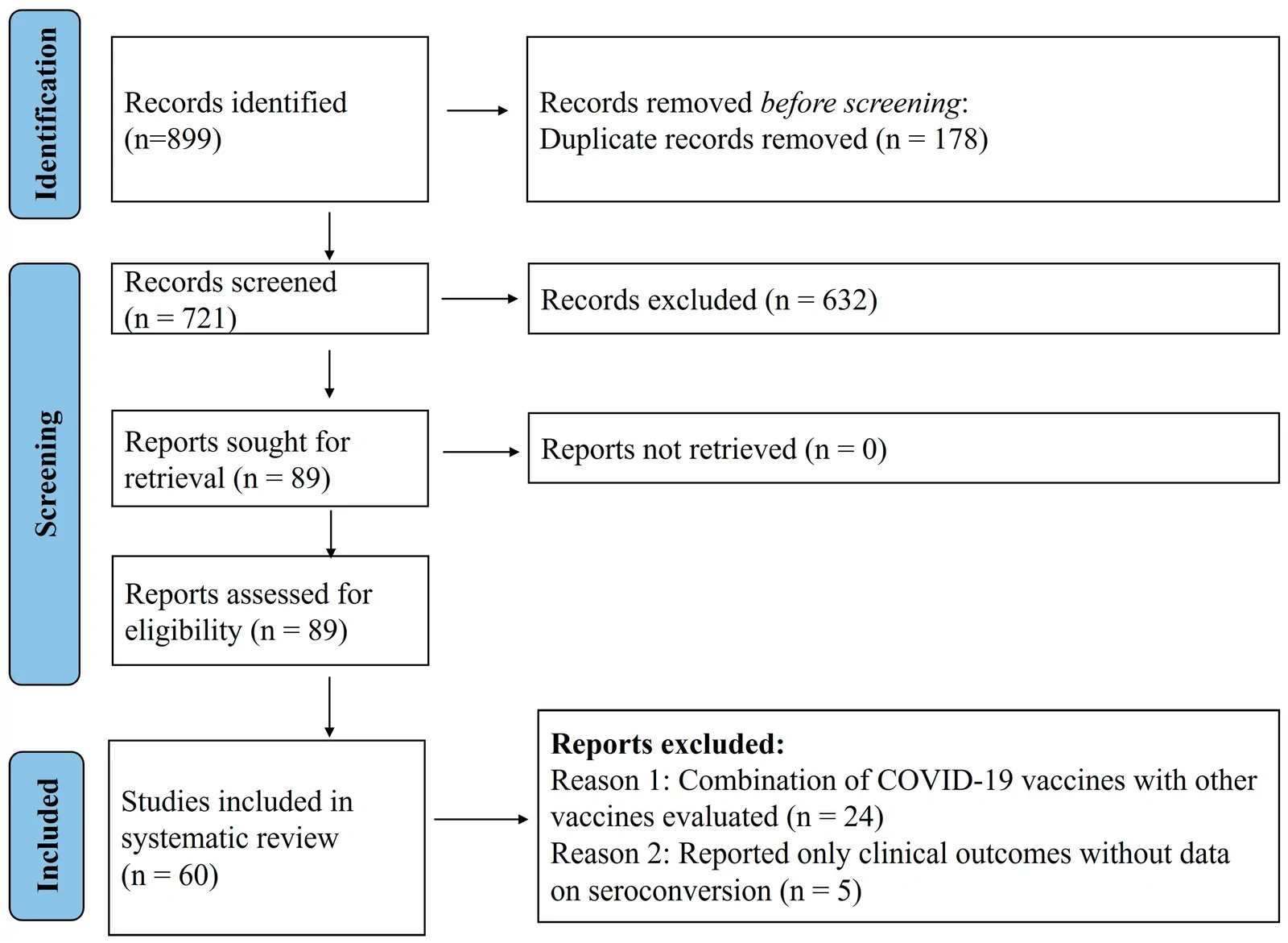
PRISMA flow diagram. A total of 60 studies were included in the systematic review from 899 records identified with the screening.

This systematic review and meta-analysis included a total of 60 studies [17,18,19,20,21,22,23,24,25,26,27,28,29,30,31,32,33,34,35,36,37,38,39,40,41,42,43,44,45,46,47,48,49,50,51,52,53,54,55,56,57,58,59,60,61,62,63,64,65,66,67,68,69,70,71,72,73,74,75,76] from an initial 899 records identified through database searching and other sources (Figure 1). The majority of studies were observational in design (n = 52), with 3 randomized controlled trials and 5 non-randomized interventional studies also identified.

All included studies involved PLWH (Electronic Supplementary Table S2). Across studies, the median age of participants was 53 years, with most patients being male. Most cohorts included individuals receiving antiretroviral therapy (ART), with varying levels of immune control, including differences in CD4+ T-cell counts and viral load suppression.

The most frequently administered vaccines were mRNA-based vaccines (BioNTech) (n = 39) and mRNA-1273 (Moderna) (n = 19), predominantly BNT162b2 (Pfizer) (n = 36). Other platforms included viral vector vaccines (n = 14) and inactivated vaccines (n = 19). Vaccination regimens ranged from one to four doses, with the two-dose (n = 42) and three-dose (n = 16) schedules being the most evaluated.

Seroconversion assessments were performed at variable time points, ranging from 10 days to over 12 months following vaccination; however, most studies (n = 44) assessed immune responses within 1–3 months after the last vaccine dose. Risk of bias assessment demonstrated that most included studies were at low to moderate risk of bias.

### 3.2. Overall Pooled Estimates

**Figure 2:**
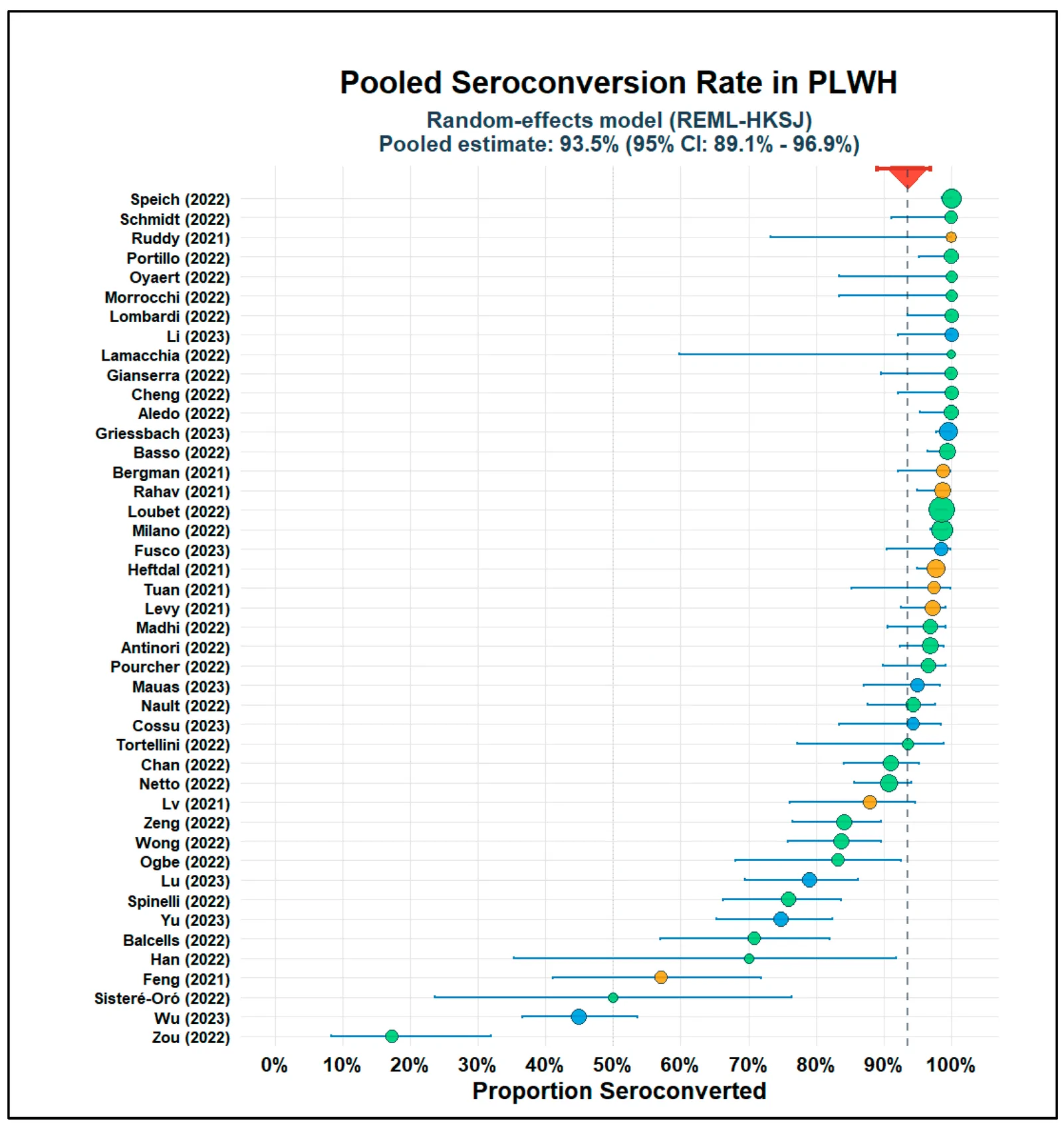
Overall seroconversion in PLWH [17,19,20,21,22,23,24,25,27,28,29,32,35,36,38,40,41,44,45,46,47,48,49,50,51,53,54,55,56,57,58,59,60,63,64,65,68,69,70,71,72,73,74,76]. The figure displays point estimates from individual studies as mentioned by circles along with the corresponding 95% confidence intervals mentioned using horizontal lines. The overall pooled estimate is mentioned using a red diamond. The color of circles represents the publication year of included studies with yellow in the year 2021, green in 2022, and blue circles in the year 2023. The size of circles is proportionate to the sample size of the respective studies. The dashed vertical blue line represents the pooled proportion.

Estimates from 44 studies comprising 5391 PLWH, the overall pooled seroconversion proportion was 93.5% (95% CI: 89.1% to 96.9%) with a prediction interval of 53.5% to 100.0% with a high heterogeneity (I^2^ = 90%) (Figure 2). Individual characteristics of studies reporting seroconversion can be found in the Electronic Supplementary Table S3. Of the total 60 studies that included in the systematic review, 13 were related to administration of combination of COVID-10 vaccines and three did not report the exact numbers of seroconversion that is amenable for pooled analysis. The remaining studies did not report seroconversion numbers that could be included in the pooling the analysis.

### 3.3. Bootstrap Analysis

**Figure 3:**
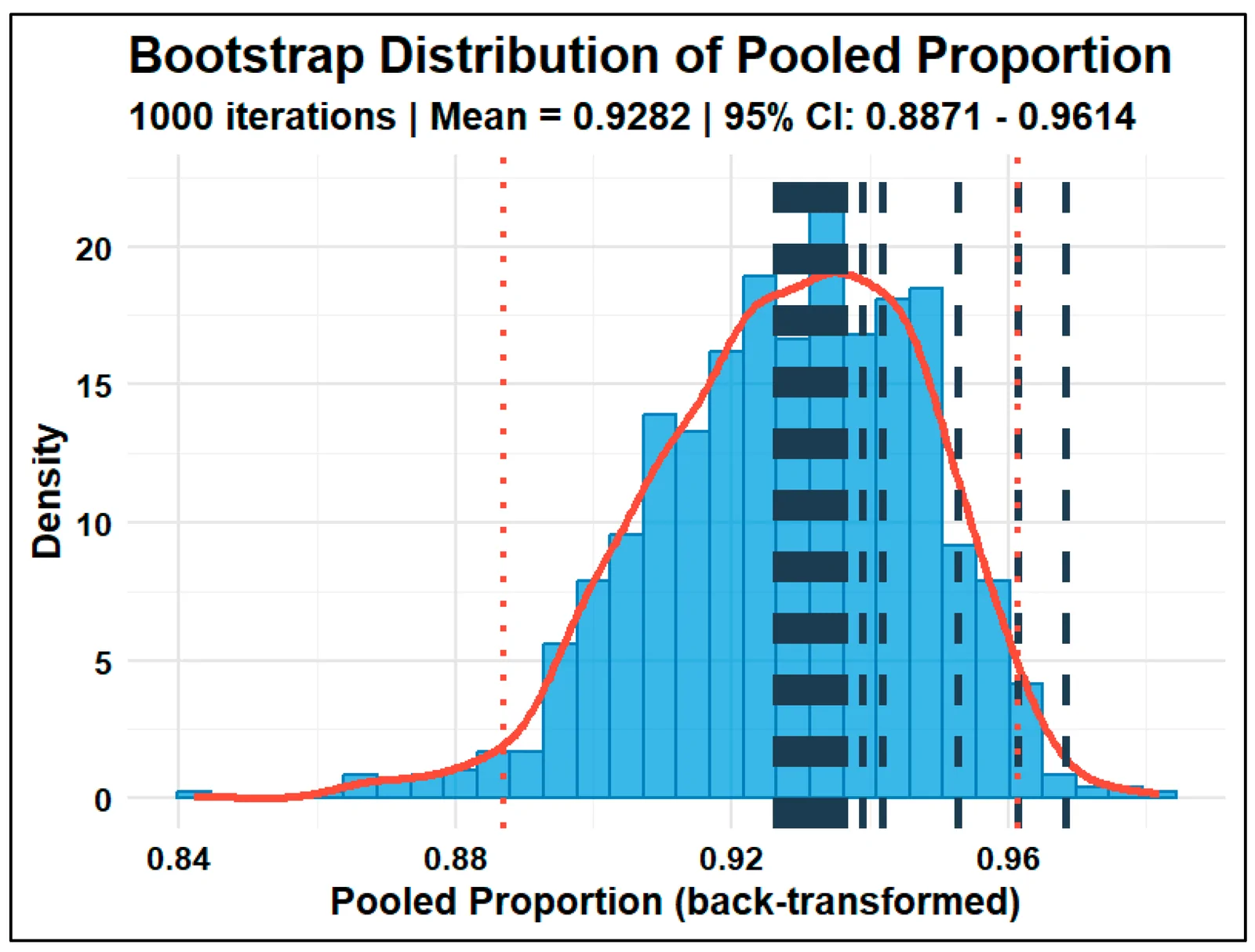
Bootstrap Distribution of the Pooled Seroconversion Proportion among PLWH following COVID-19 Vaccination. The histogram displays the distribution of pooled seroconversion proportions obtained from 1000 bootstrap resampling iterations. The red color density curve overlaid on the histogram illustrates the smooth distribution of the bootstrap estimates. The vertical black dashed line represents the original pooled proportion, and the vertical red dotted lines indicate the 95% bootstrap confidence interval boundaries.

The bootstrap distribution exhibits an approximately symmetrical pattern centered around the mean estimate of 0.9282, with the narrow spread of the distribution reflecting the minimal uncertainty in the pooled estimate despite the heterogeneity observed across individual studies (Figure 3). The close alignment between the bootstrap mean (0.9282) and the original pooled estimate (0.9348) confirms the robustness and stability of the overall estimate, providing empirical validation that the pooled proportion is not unduly influenced by any single study or small-sample bias. The relatively narrow 95% bootstrap confidence interval (0.8871 to 0.9614) indicates that the overall estimate is precise and that the findings are unlikely to change substantially with the addition of new studies, assuming they are drawn from the same underlying population.

### 3.4. Trial Sequential Analysis

**Figure 4:**
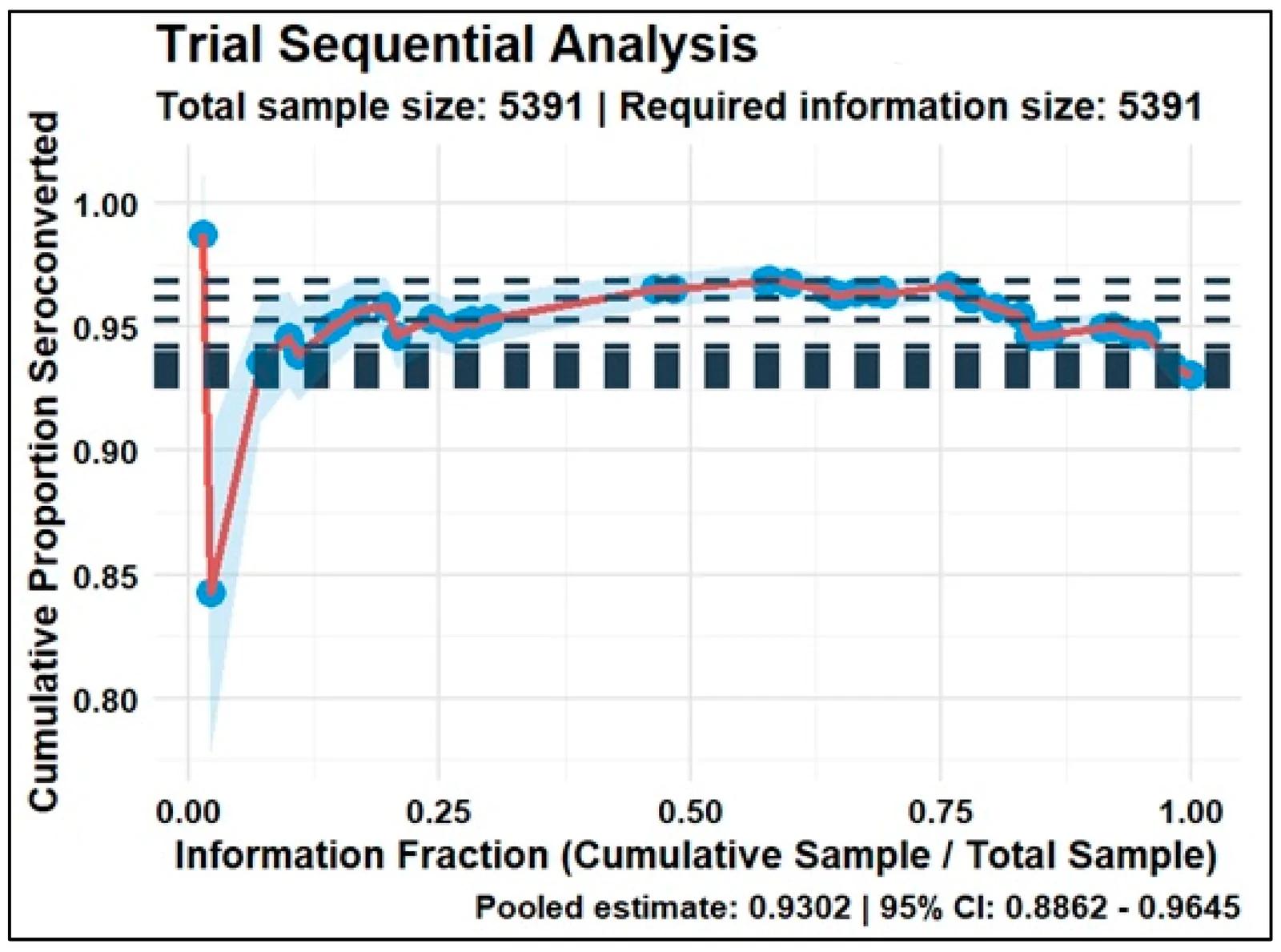
Trial Sequential Analysis of the Cumulative Evidence for Seroconversion following COVID-19 Vaccination in PLWH. The trial sequential analysis displays the cumulative pooled estimates of seroconversion proportion with addition of studies chronologically against the information fraction representing the proportion of the total sample size accumulated. The red line illustrates the cumulative pooled proportion with associated shaded confidence band representing the 95% confidence intervals at each sequential step. The black dashed horizontal line represents the final REML-HKSJ pooled proportion.

The trial sequential analysis reveals that the cumulative estimate reached relative stability after approximately 50% of the total information was accumulated, with subsequent additions of studies resulting in minimal fluctuation of the pooled proportion around the final estimate, suggesting that the evidence base achieved sufficiency for drawing reliable conclusions (Figure 4). The required information size, calculated based on the observed event proportion with alpha set at 0.05 for type I error and beta at 0.20 for type II error (80% statistical power), was met with the total sample size of 5391 participants, indicating that the accumulated evidence exceeded the minimum requirements for firm conclusions. The narrow confidence band surrounding the cumulative estimate in the latter stages of the analysis further supports the precision and stability of the findings, with the final confidence interval ranging from 0.89 to 0.97 and the prediction interval extending substantially wider from 0.53 to 1.00, highlighting the distinction between the precision of the summary estimate and the anticipated variability in future individual study results. The trial sequential analysis provides methodological reassurance that the meta-analytic findings are not driven by early extreme estimates or subject to random error due to sparse data, as the cumulative evidence trajectory demonstrates progressive convergence toward a stable pooled estimate with increasing information fraction. The crossing of the required information size threshold confirms that the available evidence is sufficient to conclude that seroconversion rates among people living with HIV following COVID-19 vaccination are consistently high, with the cumulative estimate remaining above 90% throughout the sequential analysis once adequate information was available. This analysis addresses concerns about potential random errors in cumulative meta-analyses and supports the robustness of the conclusion that PLWH generally mount robust seroconversion responses to COVID-19 vaccination, although the wide prediction interval underscores the heterogeneity in responses that may be observed in future studies or individual patient populations.

### 3.5. Sensitivity and Publication Bias Analyses

**Figure 5:**
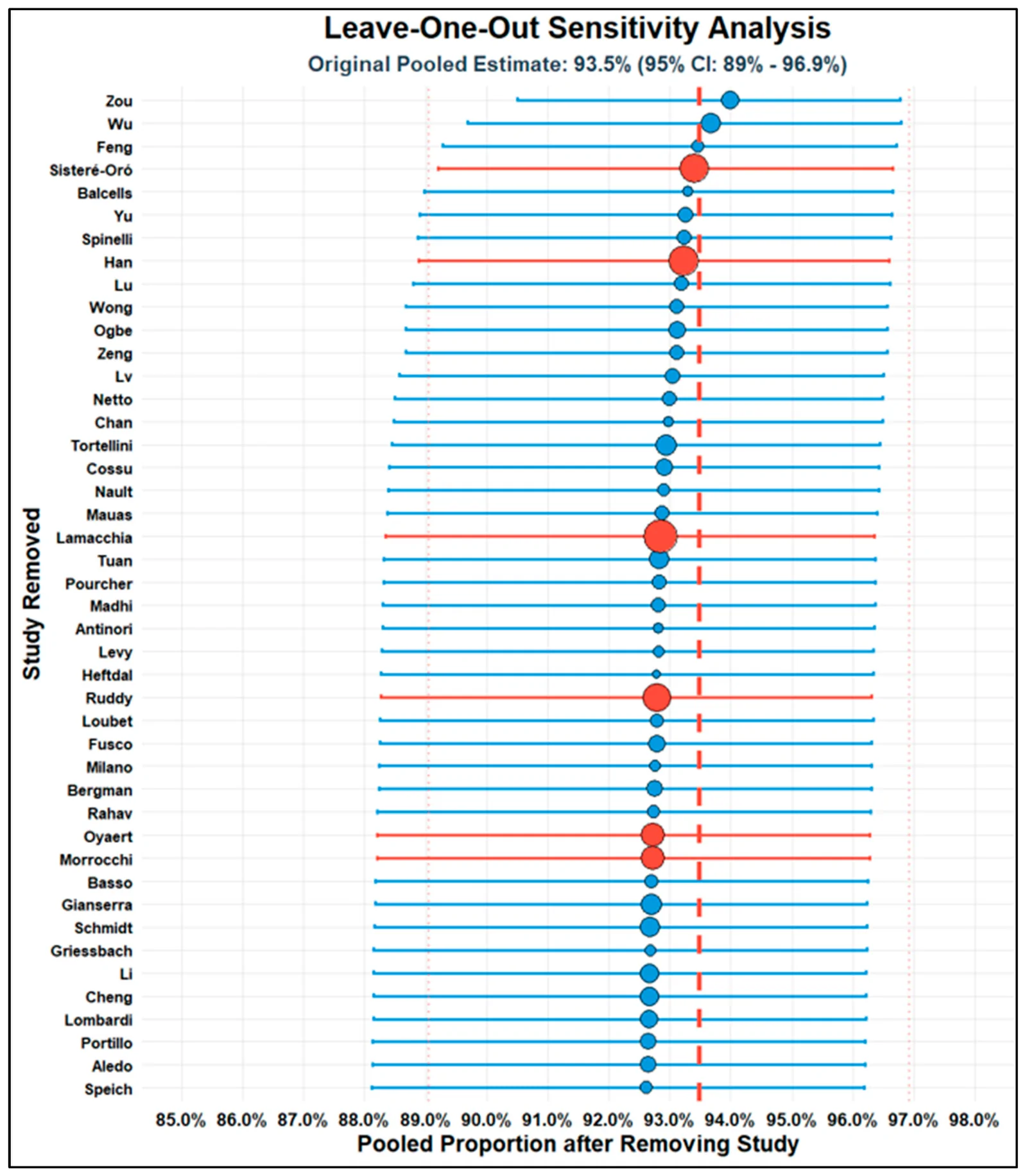
Leave-One-Out Sensitivity Analysis for the Pooled Seroconversion Proportion among PLWH following COVID-19 Vaccination [17,19,20,21,22,23,24,25,27,28,29,32,35,36,38,40,41,44,45,46,47,48,49,50,51,53,54,55,56,57,58,59,60,63,64,65,68,69,70,71,72,73,74,76]. The forest plot displays the pooled seroconversion proportion (x-axis) with circles representing the point estimate with horizontal lines representing the 95% confidence intervals for removal of estimates from the respective studies (y-axis). The vertical dashed red line represents the original pooled estimate with dotted red lines representing the original 95% confidence interval. The red circles and lines represent influential studies, and blue ones are not influential. The size of circles represents the pooled seroconversion proportion with the removal of estimates from the studies concerned.

The leave-one-out sensitivity analysis demonstrated the robustness of the pooled seroconversion estimate, as the exclusion of any single study resulted in pooled proportions ranging from 0.9262 to 0.9400, all remaining within the 95% CI of the original pooled estimate (0.8905 to 0.9693) (Figure 5). The magnitude change in the pooled proportion after removing individual studies was minimal for most studies, with 40 out of 44 studies (90.9%) showing a change of less than 1% from the original estimate of 0.9348. Four studies were identified as having a greater influence on the overall estimate, namely Lamacchia (change: 3.99%), Han (2.92%), Sisteré-Oró (2.75%), and Ruddy (2.49%), though none of these exclusions altered the pooled proportion beyond the original confidence interval boundaries or changed the direction or clinical significance of the findings. The stability of the pooled estimate across all leave-one-out iterations, combined with the narrow range of pooled proportions observed, confirms that the meta-analytic findings are not unduly driven by any single study and that the conclusion of a high seroconversion rate (approximately 93%) among PLWH following COVID-19 vaccination is robust to the exclusion of individual studies.

**Figure 6:**
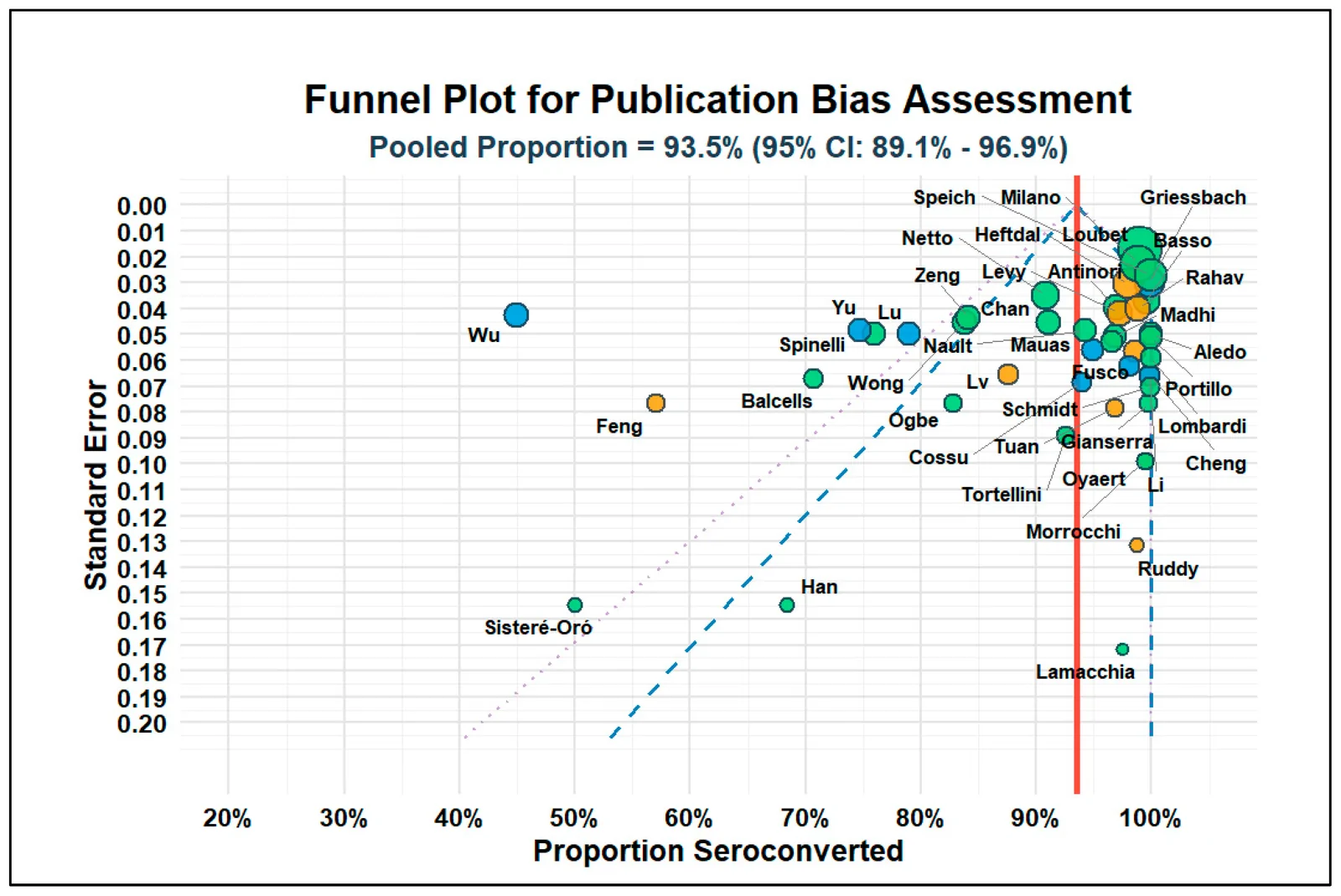
Funnel Plot for Assessment of Publication Bias in the Meta-Analysis of Seroconversion Rates following COVID-19 Vaccination among PLWH. The funnel plot depicts the seroconverted proportion on x-axis with their corresponding standard error on y-axis for each included study. The vertical solid red line represents the overall pooled proportion of 93.5% and the dashed lines indicating the 95% pseudo-confidence limits around the pooled estimate. The color of circles represents the publication year of included studies with yellow in the year 2021, green in 2022, and blue circles in the year 2023. The size of circles is proportionate to the sample size of the respective studies.

Funnel plot (Figure 6) analysis revealed asymmetrical distribution of studies, with a relative paucity of studies in the lower right quadrant, along with a statistically significant Egger’s regression test (*p* = 0.0148), indicating the presence of publication bias or small-study effects.

### 3.6. Exploratory Sub-Group Analysis

Table 1 lists the pooled estimates across various sub-groups.

#### 3.6.1. Vaccine Platform

mRNA vaccines demonstrated significantly higher pooled seroconversion rates of 98.2% (95% CI: 96.4–99.6) compared to non-mRNA vaccines at 80.5% (95% CI: 69.4–89.8), indicating a clear advantage of mRNA platforms in eliciting humoral immune responses in people living with HIV. Analysis of individual vaccines revealed BNT162b2 (97.5%, 95% CI: 94.3–99.5) and combinations of mRNA-1273 or BNT162b2 (99.7%, 95% CI: 98.8–100.0) as the most effective, while Sinopharm WIBP-CorV showed the lowest estimate of 31.0% (95% CI: 0.0–100.0), although the wide confidence intervals for several vaccine types limit definitive comparisons.

#### 3.6.2. CD4 Count

CD4 count demonstrated a graded association with seroconversion, with individuals having CD4 counts <200 cells/mm^3^ showing the lowest estimate of 53.8% (95% CI: 7.8–96.2), those with CD4 counts of 200–500 cells/mm^3^ showing 86.2% (95% CI: 58.3–100.0), and those with >500 cells/mm^3^ showing 93.5% (95% CI: 87.5–97.8), while individuals with >300 cells/mm^3^ showed the highest estimate of 99.4% (95% CI: 93.4–100.0), suggesting that preserved immune function is essential for optimal vaccine responses.

#### 3.6.3. ART Status

ART status revealed a stark contrast, with PLWH on ART showing a pooled estimate of 93.5% (95% CI: 88.6–97.3) compared to those not on ART at 49.6% (95% CI: 0.0–100.0), emphasizing the critical role of ART in facilitating robust vaccine responses.

#### 3.6.4. Prior COVID-19 Infection

Prior COVID-19 infection was associated with significantly higher seroconversion rates, with studies including participants with prior infection showing a pooled estimate of 99.1% (95% CI: 97.2–100.0) compared to 89.5% (95% CI: 83.3–94.5) among those without prior infection, suggesting that natural immunity combined with vaccination produces near-universal seroconversion.

#### 3.6.5. Seroconversion Assays

The subgroup analyses revealed substantial variability in seroconversion rates across different assay types, with the highest pooled estimates observed for Roche assays (100.0%, 95% CI: 99.8–100.0) and Abbott assays (98.2%, 95% CI: 71.3–100.0), while neutralizing antibody assays demonstrated the lowest estimate (69.8%, 95% CI: 23.4–99.9), although confidence intervals were wide for several assay categories indicating imprecision in the estimates.

#### 3.6.6. Seroconversion Definitions

Seroconversion definitions varied considerably across studies, with stricter criteria such as IgG ≥ 33.8 BAU/mL reported in six studies yielding estimates of 98.9% (95% CI: 95.4–100.0) and IgG ≥ 0.8 U/mL showing 100.0% (95% CI: 96.8–100.0), whereas more lenient or alternative definitions such as IgG ≥ 0.18 EU/mL produced substantially lower estimates of 31.0% (95% CI: 0.0–100.0), highlighting the critical impact of assay cutoff selection on reported seroconversion rates.

#### 3.6.7. Study Designs

Observational studies yielded a pooled estimate of 91.3% (95% CI: 86.0–95.5) compared to clinical trials at 97.9% (95% CI: 91.8–100.0), indicating that controlled trial settings may produce higher seroconversion rates than real-world observational data.

#### 3.6.8. Number of Vaccine Doses

The number of vaccine doses showed comparable estimates between two doses (92.2%, 95% CI: 86.7–96.5) and three doses (91.7%, 95% CI: 80.1–98.9), suggesting that the incremental benefit of a third dose may be modest in this population.

The estimates for certain subgroups, particularly PLWH not on ART (49.6%, 95% CI: 0.0–100.0) and those with CD4 counts below 200 cells/mm^3^ (53.8%, 95% CI: 7.8–96.2), should be interpreted with caution due to the limited number of studies and participants contributing to these analyses. The confidence intervals reaching the boundary of 0.0% for the non-ART subgroup reflect the instability inherent in meta-analyzing proportions when event rates are extreme and sample sizes are small. Although we employed the Freeman-Tukey double arcsine transformation, a variance-stabilizing approach specifically recommended for meta-analyses of proportions, this transformation cannot eliminate the uncertainty introduced by sparse data. These findings highlight critical knowledge gaps and underscore the urgent need for larger, dedicated studies in these high-risk subgroups. Heterogeneity as measured by the I^2^ statistic varied across subgroups, with some categories showing I^2^ values approaching or reaching 100% indicating substantial between-study variability, while others demonstrated low heterogeneity suggesting consistent findings across studies. Overall, these subgroup findings underscore the multifactorial nature of seroconversion responses in PLWH, with vaccine type, prior infection status, CD4 count, and ART status emerging as the most influential determinants of humoral immune response to COVID-19 vaccination. The wide confidence intervals observed across multiple subgroups, particularly those with limited numbers of studies, highlight the need for additional research to refine these estimates and confirm the observed patterns.

### 3.7. Safety Profile of COVID-19 Vaccines in PLWH

Among the 60 included studies, vaccine safety data were reported in 18 studies. Key details of safety profile amongst the included studies can be found in the Electronic Supplementary Table S4. Overall, COVID-19 vaccines demonstrated a favorable safety profile in PLWH. Local and systemic adverse events were predominantly mild to moderate in severity. The most frequently reported local reaction was injection-site pain (median rate: 23.8%), while common systemic adverse events included fatigue (median: 6.4%), headache (median: 6.4%), and myalgia (median: 5.05%). Importantly, serious adverse events were rare, and no consistent association was observed between vaccination and HIV disease progression. Across studies, no significant changes in CD4+ T-cell counts or viral load were observed following vaccination. Most studies explicitly reported no increase in HIV-related complications, hospitalization, or vaccine-related mortality. Several studies reported that COVID-19 vaccination was well tolerated in the PLWH group. Collectively, these findings support the overall safety and tolerability of COVID-19 vaccines in PLWH, despite variability in immunogenicity across immune statuses.

### 3.8. Effect of COVID-19 Vaccination on CD4+ T-Cell Counts

Among the included studies, six studies evaluated the effect of COVID-19 vaccination on HIV-specific disease markers, specifically absolute CD4+ T cell count. For instance, absolute CD4+ T-cell counts remained relatively stable after vaccination, although moderate fluctuations were also observed. For example, Feng et al. reported a decrease in mean CD4+ count from 659 ± 222 cells/μL before vaccination to 477 ± 151 cells/μL after vaccination [28]. Similarly, Levy et al. documented a reduction in geometric mean CD4 count from 700 cells/μL pre-vaccination to 634 after the second dose [45]. Aledo et al. observed only a minimal median change of 17 cells/μL between pre- and post-vaccination measurements [64]. Contrarily, Gianserra et al. documented an increase in median CD4 count from 687 cells/mm^3^ before vaccination to 732 cells/mm^3^ post-booster dose [29]. Likewise, Hassold et al. observed a rise in median CD4+ count from 386 cells/µL after the primary 2-dose vaccination to 556 cells/µL post-booster, while Fusco et al. reported comparable findings by reporting that the median CD4 count increased after the third dose, as it was 663 cells/μL before vaccination and 707 cells/μL after the third dose [27,34]. Collectively, these findings indicated that although transient fluctuations in CD4+ counts were observed following COVID-19 vaccination, there was no consistent pattern of clinically significant CD4+ decline across the included studies.

## 4. Discussion

This systematic review and meta-analysis demonstrate that COVID-19 vaccination induces a robust humoral immune response in most PLWH. The comprehensive analysis of this study produced a pooled seroconversion rate of 93.5%, indicating that most PLWH develop a measurable humoral response following COVID-19 vaccination. This meta-analysis confirms our primary hypothesis that COVID-19 vaccination induces robust seroconversion rates (93.5%) in most PLWH. Our secondary hypotheses were also largely confirmed: mRNA vaccines demonstrated superior immunogenicity compared to non-mRNA platforms (98.2% vs. 80.5%); seroconversion rates exhibited a graded association with CD4+ T-cell counts (53.8% for CD4 < 200 vs. 93.5% for CD4 > 500 cells/mm^3^); ART use and prior SARS-CoV-2 infection were associated with significantly higher responses (93.5% vs. 49.6% and 99.1% vs. 89.5%, respectively); and the safety profile was favorable without evidence of HIV disease progression. However, the wide confidence intervals for certain subgroups highlight important knowledge gaps requiring further investigation. This result is consistent with other meta-analyses, including Zhao et al., which reported a seroconversion rate of 93.7% in PLWH after the second vaccine dose, compared with 97.6% in healthy controls [12]. Søndergaard et al. likewise reported that seroconversion among PLWH receiving non-inactivated COVID-19 vaccines reached 91.62% [10].

Historically, reduced responses to various vaccines, chronic immune activation, and impaired adaptive immunity have placed PLWH among patient groups expected to be highly vulnerable to COVID-19 infection. In contrast, healthy individuals almost consistently achieve seroconversion rates of approximately 100% after two doses of the COVID-19 vaccine, whereas other immunocompromised groups also show diminished responses [14]. A recent meta-analysis of other immunocompromised groups, particularly solid organ transplant recipients, showed lower COVID-19 vaccination response rates, with an overall pooled seroconversion rate of 49% [16]. A similar pattern was therefore anticipated for PLWH. However, this meta-analysis indicates that immunocompetence can be achieved in PLWH when CD4 T cell function is preserved, effective antiretroviral therapy is maintained, and an appropriate vaccine type is used. These factors are major determinants of vaccine response, as shown by the analysis. Maintaining immunocompetence after vaccination in PLWH is critical, given their increased risk of severe COVID-19, hospitalisation, and mortality. A high seroconversion rate suggests that vaccination provides substantial immune protection in this vulnerable population, thereby supporting current recommendations for COVID-19 vaccination in PLWH [77].

In this study, a comprehensive subgroup analysis was conducted to examine differences in COVID-19 vaccine effectiveness across vaccine types. The findings of this analysis indicate that mRNA vaccines induce a superior humoral response in PLWH, as evidenced by a pooled seroconversion rate of 98% in this group. In contrast, non-mRNA vaccines were substantially less effective, with a pooled seroconversion rate of 80.5%. Cheng et al. also support this finding, reporting an immune response rate (IRR) of 98.91% for mRNA vaccines, compared to 92.48% for inactivated vaccines [8]. Søndergaard et al. further noted that inactivated vaccines yielded the lowest seroconversion rates; excluding these vaccines increased seroconversion from 87% to 91.62% [10]. Additionally, Zhou et al. also reported a pooled seroconversion rate of 96.1% among PLWH receiving mRNA vaccines, compared with 59.0% among those receiving inactivated vaccines [13]. The Individual vaccine subgrouping of this analysis showed that CoronaVac and BBIBP-CorV induced immune responses of approximately 81.6% and 70%, respectively. Contrastingly, mRNA vaccines consistently achieved higher seroconversion rates, with 98% reported for BNT162b2 or mRNA-1273 regimens. Hence, mRNA vaccines elicited superior immune responses than non-mRNA vaccines in PLWH, which supports their preferential use in PLWH when available. Mechanistically, mRNA vaccines induce endogenous spike antigen synthesis in host cells, permitting efficient antigen presentation through both MHC class I and class II pathways. This process coordinates the activation of CD8 cytotoxic and CD4 helper T cells, including Th1 responses characterised by interferon-γ production. Cross-presentation by professional antigen-presenting cells further amplifies cellular immunity. Enhanced T cell help facilitates germinal centre formation, B cell maturation, and antibody production. These mechanisms provide a biologically plausible explanation for the higher seroconversion rates observed with mRNA vaccines [78].

Comparatively, inactivated vaccines contain non-replicating viral particles that are processed through the exogenous antigen presentation pathway, leading predominantly to MHC class II-mediated activation of CD4 helper T cells and subsequent antibody production. Although these vaccines can induce a neutralising IgG response, they are generally less likely to induce a strong CD8 T cell response due to limited intracellular antigen expression and inefficient MHC class I presentation. Reduced cellular immune stimulation may result in weaker T cell help for germinal centre reactions, memory B cell generation, and affinity maturation, thereby contributing to lower seroconversion rates [79].

CD4 count is another major determinant of humoral response in PLWH. Among all subgroup analyses performed in this study, CD4 count showed one of the clearest biological gradients: patients with a CD4 count below 200 had a seroconversion rate of 53.8%, which was markedly lower than the 93.4% rate observed in those with a CD4 count above 500. These findings are supported by other studies, such as Farhadian et al., who reported a pooled seroconversion rate of 98.4% for patients with CD4 > 500 cells/mm^3^ and 75.2% for patients with CD4 < 200 cells/mm^3^ after two doses of an mRNA vaccine [7]. Additionally, Kang et al. reported that the seroconversion rate in patients with CD4 counts ≥500 cells/µL could reach 97.99% after a second COVID-19 vaccine dose, compared with a lower rate of 91.44% in patients with a CD4 count < 500 cells/µL [9]. Following viral infection or vaccination, CD4 T cells coordinate multiple aspects of the adaptive immune response. They recruit immune cells to secondary lymphoid tissues and sites of infection, facilitate interactions between dendritic cells and CD8 T cells, and promote the migration of innate and antigen-specific effector cells to infected tissues. CD4 T cells are indispensable for the expansion, maintenance, and long-term memory formation of CD8 T cells, particularly during chronic viral infections, where sustained cytokine production is essential to preserve CD8 T cell function. Moreover, specialised follicular helper T cells provide significant assistance to B cells by generating high-affinity neutralising antibody responses. Consequently, reduced CD4 T cell numbers in PLWH may compromise the immune response and lower seroconversion rates relative to healthy controls [80].

This synthesis supports the evidence that PLWH with advanced immunosuppression and low CD4 counts remain at increased risk of inadequate immune responses after vaccination. Therefore, these individuals should be prioritised for enhanced preventive strategies, including additional vaccine doses, pre-exposure prophylaxis, optimised booster schedules, and close clinical monitoring to ensure protection against severe COVID-19. Further research is required to investigate PLWH with severe immunosuppression. Although subgroup analysis suggested a low seroconversion rate among patients with low CD4 T cells, the wide confidence intervals reflect limited sample size and considerable uncertainty in the pooled estimate. Large prospective studies evaluating cellular and humoral immune responses, durability of protection, and clinical effectiveness are needed to guide evidence-based vaccination recommendations for this high-risk population.

Antiretroviral therapy has revolutionised HIV treatment, and the immune restoration following ART has dramatically reduced the incidence and severity of opportunistic diseases and mortality. Findings indicate that patients receiving ART have a substantially higher seroconversion rate than those not receiving ART. In this study, patients taking ART achieved a seroconversion rate of 93.5%, compared with 49.5% among patients not reported to be taking it. However, most comprehensive meta-analyses did not provide a formal subgroup analysis for patients taking ART. The study by Cheng et al. reported that patients on ART achieved a seroconversion rate of 97.2% after receiving a booster COVID-19 vaccination, which was almost equivalent to that of healthy controls [8]. Adherence to ART is a key determinant of vaccine immunogenicity in PLWH, as it maintains viral suppression and preserves immune function. Most patients in the included studies received contemporary ART regimens comprising nucleoside/nucleotide reverse transcriptase inhibitors (NRTIs), non-nucleoside reverse transcriptase inhibitors (NNRTIs), protease inhibitors (PIs), and/or integrase strand transfer inhibitors (INSTIs). These agents suppress HIV replication by inhibiting reverse transcription and viral DNA integration into the host genome, thereby preserving CD4 T cell counts and function and reducing chronic immune activation. Given the importance of CD4 T cells in coordinating an effective immune response, sustained ART adherence is strongly supported by evidence and helps create a more favourable immunological environment for COVID-19 vaccination, thereby increasing seroconversion rates. Non-compliance with these medications can significantly blunt the immune response, lead to failure to maintain CD4 counts, and increase the risk of severe COVID-19 infection in PLWH [81].

Collectively, the findings of this meta-analysis indicate a heightened need to optimise antiretroviral therapy coverage, with adherence being essential not only for sustaining HIV viral suppression and immune restoration but also for maximising the vaccine-induced immune response. The mechanism of action of ART restores immune system function, thereby protecting PLWH against vaccine-preventable infections such as SARS-CoV-2 and other opportunistic infections, reducing morbidity, and improving long-term health outcomes. This review identified substantial methodological variation, with considerable variability in reported seroconversion rates depending on assay type and seroconversion definition. Most studies utilising the Roche and Abbott binding antibody assays consistently reported near-complete seroconversion (100% and 98.2%, respectively), whereas neutralising antibody assays yielded considerably lower estimates. These differences reflect fundamental distinctions in assay sensitivity, antibody targets, and the measurement of antibody presence versus functional neutralising capacity.

Similarly, there was marked variation across seroconversion thresholds. The threshold of IgG ≥ 33.8 BAU/mL was among the most frequently utilised definitions in the included studies and is consistent with the World Health Organisation (WHO) international standardisation framework. These findings strongly support the adoption of a standardised seroconversion threshold, particularly the WHO-calibrated BAU/mL-based definitions, for future research on vaccine immunogenicity in PLWH. Standardisation offers advantages including improved comparability across studies, more robust evidence synthesis, and enhanced reproducibility.

Furthermore, patients with prior SARS-CoV-2 infection exhibited higher seroconversion rates than those without prior infection. The results of this analysis showed that patients with prior infection had a pooled seroconversion rate of 99%, compared to 89.5% in patients without prior infection. An extensive meta-analysis by Zhou et al. also reported similar findings: studies of patients with no history of COVID-19 infection reported a pooled seroconversion rate of 87.7%, compared with 94.9% among patients who might have had prior exposure to the virus [13].

PLWH with hybrid immunity consistently demonstrated the highest levels of vaccine-induced humoral responses. These results suggest that repeated antigenic exposure through natural infection and vaccination may, at least partially, overcome the immunologic deficit associated with chronic HIV infection. Hybrid immunity refers to the immune state that arises in patients who have experienced SARS-CoV-2 infection and subsequently received vaccination, leading to an enhanced immune response. Mechanistically, this synergy is induced by priming the B and T cell repertoire during natural infection, followed by potent affinity maturation and clonal expansion after vaccination. Hybrid immunity leads to a higher and more durable anti-spike and anti-receptor binding domain (RBD) IgG response that persists for months. Additionally, hybrid immunity promotes broad cross-variant recognition, including improved recognition of antigenically divergent variants such as Omicron, due to enhanced targeting of conserved epitopes and increased Fc receptor binding antibody function that facilitates viral clearance. These findings have crucial implications for personalising vaccination strategies in PLWH, as infection history can help identify individuals more likely to achieve a robust immune response and those who might benefit from an additional vaccine dose or closer immunological monitoring [82]. Vaccination should remain a priority for all PLWH, regardless of prior infection status. Although prior infection may enhance vaccine immunogenicity, the durability of these antibody responses in PLWH remains uncertain. Persistent immune dysregulation, chronic inflammation, and variable CD4 T cell reconstitution could influence the long-term maintenance of both humoral and cellular immunity. Longitudinal studies and further investigations are needed to evaluate the persistence and clinical protection of hybrid immunity.

This comprehensive synthesis supports current recommendations advocating COVID-19 vaccination among PLWH, particularly those receiving antiretroviral therapy with preserved CD4 T cell counts. These findings are reassuring, as they indicate that the majority of PLWH can mount a measurable humoral immune response following vaccination. However, seroconversion represents only one aspect of vaccine-induced immunity and does not reflect long-term protection against SARS-CoV-2 infection, severe disease, or clinical effectiveness. Antibody magnitude, neutralising capacity, durability, and vaccine-induced cellular immunity contribute substantially to protection. Waning immunity over time and the continuous emergence of immune-evasive SARS-CoV-2 variants could disproportionately affect PLWH, particularly those with incomplete immune reconstitution. Ongoing surveillance of vaccine effectiveness, including longitudinal assessment of cellular and humoral immune responses, remains essential. Despite these limitations, our analysis of the factors associated with seroconversion remains crucial, as it provides valuable insights into the determinants of vaccine responsiveness in PLWH and, hence, helps identify subgroups that may need individualised vaccination approaches.

Moreover, this study demonstrates that COVID-19 vaccines have a reassuring safety profile among people living with HIV (PLWH). Across all studies reporting safety outcomes, the adverse effects were predominantly mild to moderate and consistent with the reactogenicity described in the general population. Injection site pain is the most common local reaction, while fatigue, headache, and myalgia were the most frequently reported systemic adverse events, which all occur at relatively low median rates. Most significantly, serious vaccine-related adverse events were not very common, and there is no evidence that suggests an increased risk of HIV disease progression following vaccination. Therefore, the consistent observation of the stable immunological and virological parameters, in addition to the absence of increased HIV related complications, hospitalizations, or vaccine-related mortality, provides a strong reassurance regarding the tolerability of the present or current COVID-19 vaccines for PLWH. Hence, concerns regarding vaccine safety should not constitute a barrier to vaccination in PLWH, even though differences in immunogenicity could persist in patients suffering from more advanced immunosuppression. The absence of a consistent decline in CD4+ T-cell counts following COVID-19 vaccination across the six studies that evaluated this outcome provides reassurance that vaccination does not adversely affect HIV disease progression. Although transient fluctuations were observed in some individuals, these variations lacked a consistent pattern and were not clinically significant, further supporting the overall safety profile of COVID-19 vaccines in PLWH.

## 5. Strengths, Limitations and Future Directions

This systematic review and meta-analysis possess several notable strengths that enhance the validity and clinical utility of its findings. The comprehensive literature search across multiple databases without language or year restrictions, coupled with the prospective registration of the protocol, ensures methodological rigor and transparency. The application of the Freeman-Tukey double arcsine transformation with REML estimation and HKSJ adjustment represents a statistically robust approach for pooling proportions, particularly given the expected heterogeneity across studies. The extensive subgroup analyses examining nine clinically relevant variables, including CD4 count, ART status, vaccine platform, and prior infection status, provide granular insights into the determinants of seroconversion that are directly applicable to clinical decision-making. The internal validation through bootstrap resampling with 1000 iterations, the trial sequential analysis confirming sufficiency of evidence, and the comprehensive sensitivity analyses collectively strengthen confidence in the robustness and stability of the pooled estimates. The inclusion of both immunogenicity and safety outcomes offers a balanced assessment of the benefit-risk profile of COVID-19 vaccination in this vulnerable population.

Several limitations warrant consideration when interpreting these findings. The substantial heterogeneity observed across studies, despite extensive subgroup analyses, suggests the presence of unmeasured sources of variability that could affect the generalizability of the findings, including differences in study populations, geographic settings, timing of antibody assessment, and baseline participant characteristics. The statistically significant publication bias detected by Egger’s test indicates that smaller studies with lower seroconversion rates may be underrepresented in the published literature, potentially inflating the overall pooled estimate. The predominant inclusion of studies from high-income countries limits the generalizability of findings to low- and middle-income settings, where the burden of HIV is highest and non-mRNA vaccines are more commonly deployed. The variability in seroconversion definitions and assay types across studies complicates direct comparisons and highlights the need for standardized thresholds in future research. The limited number of studies in several subgroup categories, particularly for patients not on ART and those with CD4 counts below 200 cells/mm^3^, resulted in wide confidence intervals and imprecise estimates that preclude definitive conclusions. Furthermore, the focus on seroconversion as a binary outcome does not capture the full spectrum of vaccine-induced immunity, including neutralizing antibody titers, T-cell responses, and the durability of protection over time.

Future research should prioritize several key areas to address the gaps identified in this meta-analysis. Large prospective studies with extended longitudinal follow-up are urgently needed to evaluate the durability of antibody responses, the kinetics of waning immunity, and the correlates of protection against emerging SARS-CoV-2 variants in PLWH, particularly those with advanced immunosuppression. Investigations incorporating cellular immunity assessments, including CD4 and CD8 T-cell responses, would provide a more comprehensive understanding of vaccine-induced protection beyond humoral responses alone. Studies specifically focused on PLWH with CD4 counts below 200 cells/mm^3^ and those not receiving ART are critically needed to refine estimates and guide vaccination strategies for these high-risk subgroups, as current evidence remains limited and imprecise. Research evaluating vaccine effectiveness rather than immunogenicity, particularly against severe COVID-19 outcomes, hospitalization, and mortality, would provide direct clinical evidence to support vaccination recommendations. The conduct of studies in low- and middle-income countries where inactivated and viral vector vaccines are predominantly used is essential to ensure global applicability of findings and to address health equity considerations. Investigation into optimal vaccination schedules, including the role of heterologous boosting, additional doses, and the timing of vaccinations relative to ART initiation or immune reconstitution, could further optimize vaccine responses. Finally, future meta-analyses should incorporate standardized reporting frameworks for seroconversion definitions and assay types to facilitate more robust evidence synthesis and cross-study comparisons, thereby strengthening the evidence base for clinical and public health decision-making in this vulnerable population.

## 6. Conclusions

This meta-analysis confirms that COVID-19 vaccination is both immunogenic and safe in PLWH, reinforcing current recommendations for universal vaccination in this population. Optimizing antiretroviral therapy coverage, prioritizing mRNA vaccines where available, and implementing tailored booster strategies for those with low CD4 counts are key clinical priorities. However, publication bias, substantial heterogeneity, and wide prediction intervals highlight the need for ongoing surveillance of vaccine effectiveness and durability, particularly against emerging variants. Future research should focus on longitudinal immune assessments, clinical effectiveness, and optimizing responses in vulnerable subgroups, including those with advanced immunosuppression and those in low-resource settings. Ultimately, achieving optimal vaccine responses requires a multifaceted approach integrating effective ART, immune preservation, and individualized vaccination strategies.

## Figures and Tables

**Table 1:** Pooled Estimates in Sub-groups.

Variable	Subgroup	Number of Studies	Total Number of PLWH	Pooled Proportion of Seroconversion	Lower Limit of 95% CI for the Pooled Proportion	Upper Limit of 95% CI for the Pooled Proportion	I^2^-Value
Assay type	ARCHITECT	3	160	97.4	68.7	100.0	0.8
	ELISA	15	2109	84.5	69.2	95.5	1
	Diasorin	2	388	96.2	0	100.0	0.9
	Roche	6	806	100.0	99.8	100.0	0
	sVNT	5	345	82.7	61.2	96.9	0.9
	CLIA	13	656	96.2	90.1	99.7	0.9
	Abbott	2	581	98.2	71.3	100.0	0.4
	Neutralizing antibody assay	5	107	69.8	23.4	99.9	0.9
Prior COVID-19 infection status	No	43	3495	89.5	83.3	94.5	0.9
	Some	8	1290	99.1	97.2	100.0	0.7
Seroconversion definition	IgG ≥ 7.1 BAU/mL	4	250	97.4	86.1	100.0	0.7
	IgG ≥ 33.8 BAU/mL	6	457	98.9	95.4	100.0	0.5
	IgG ≥ 0.8 U/mL	4	434	100.0	96.8	100.0	0.4
	≥4-fold rise in titre	2	154	98.8	36.9	100.0	0.5
	≥15 AU/mL	2	246	96.5	0	100.0	0.9
	IgG titre ≥ 1.1	5	1341	99.0	97.9	99.7	0.1
	Positive IgG	8	243	85.7	56.8	100.0	0.9
	sVNT inhibition > 20%	4	181	83.8	51.1	100.0	0.9
	sVNT reciprocal titer ≥ 10	2	100	79.0	0	100.0	0.6
	IgG ≥ 0.18 EU/mL	2	184	31.0	0	100.0	0.9
	S/CO value ≥ 1.0	4	189	94.1	71.8	100.0	0.8
Vaccine type	mRNA	32	4009	98.2	96.4	99.6	0.8
	non-mRNA	24	1382	80.5	69.4	89.8	0.9
CD4 count (cells/mm^3^)	<200	4	94	53.8	7.8	96.2	0.9
	200–500	6	459	86.2	58.3	99.9	1
	>500	26	2233	93.5	87.5	97.8	0.9
	>300	6	515	99.4	93.4	100.0	0.7
	≤300	3	65	89.9	7.9	100.0	0.9
	>200	2	53	85.0	82.0	87.8	0
Study design	Observational	49	4521	91.3	86.0	95.5	0.9
	Clinical trial (or post hoc from clinical trials)	7	870	97.9	91.8	100.0	0.8
Individual vaccines	BNT162b2 or mRNA-1273	4	174	98.3	85.8	100.0	0.6
	CoronaVac	5	483	81.6	61.9	95.4	0.9
	BNT162b2	20	1923	97.5	94.3	99.5	0.8
	BBIBP-CorV	2	107	70.5	0	100.0	0.9
	mRNA-1273 or BNT162b2	5	1710	99.7	98.8	100.0	0.6
	mRNA-1273	3	202	96.3	68.4	100.0	0.8
	CoronaVac or BBIBP-CorV	2	58	88.1	0	100.0	0.6
	Sinopharm WIBP-CorV	2	184	30.9	0	100.0	0.9
	CoronaVac or Covilo	2	57	100.0	99.7	100.0	0
	CoronaVac or others	5	107	69.8	23.4	99.9	0.9
Number of doses	Two	39	4259	92.2	86.7	96.5	0.9
	Three	15	1018	91.7	80.1	98.9	0.9
ART status	Yes	41	3625	93.5	88.6	97.3	0.9
	No	2	29	49.6	0	100.0	0.9

ART, antiretroviral therapy; AU/mL, arbitrary units per millilitre; BAU/mL, binding antibody units per millilitre; CI, confidence interval; CLIA, chemiluminescence immunoassay; ELISA, enzyme-linked immunosorbent assay; EU/mL, ELISA units per millilitre; IgG, immunoglobulin G; mRNA, messenger ribonucleic acid; PLWH, people living with HIV; S/CO, signal-to-cutoff ratio; sVNT, surrogate virus neutralisation test. Studies may appear in multiple subgroups across different variables (such as vaccine type, CD4 count, ART status) if they reported data for multiple categories. For each subgroup, studies were disaggregated based on the specific variable of interest. I^2^ values represent the proportion of total variation across studies attributable to heterogeneity. Estimates with confidence intervals extending to 0.0% or 100.0% should be interpreted with caution due to limited sample sizes.

## Data Availability

The data presented in this study are available on request from the corresponding author. The data was obtained from published studies that are copyrighted to the publishers and data cannot be publicly displayed.

## Supplementary Materials

The following supporting information can be downloaded at: https://www.mdpi.com/article/10.3390/vaccines14080648/s1, Table S1. Search strategy in PubMed; Table S2. Summary of Demographic Characteristics of Included Studies; Table S3. Summary of Serological Characteristics of Included Studies; Table S4. Summary of Safety Characteristics of Included Studies.

## Abbreviations

AIDS	Acquired Immunodeficiency Syndrome
ART	Antiretroviral Therapy
BAU/mL	Binding Antibody Units per Millilitre
CDC	Centers for Disease Control and Prevention
CD4	Cluster of Differentiation 4
CD8	Cluster of Differentiation 8
CENTRAL	Cochrane Central Register of Controlled Trials
CI	Confidence Interval
COVID-19	Coronavirus Disease 2019
DNA	Deoxyribonucleic Acid
EU/mL	ELISA Units per Millilitre
FDA	Food and Drug Administration
HIV	Human Immunodeficiency Virus
HKSJ	Hartung–Knapp–Sidik–Jonkman
IgG	Immunoglobulin G
INSTI	Integrase Strand Transfer Inhibitor
IRR	Immune Response Rate
JBI	Joanna Briggs Institute
MHC	Major Histocompatibility Complex
mRNA	Messenger Ribonucleic Acid
NNRTI	Non-Nucleoside Reverse Transcriptase Inhibitor
NRTI	Nucleoside/Nucleotide Reverse Transcriptase Inhibitor
PI	Protease Inhibitor
PLWH	People Living with HIV
PRISMA	Preferred Reporting Items for Systematic Reviews and Meta-Analyses
RBD	Receptor Binding Domain
RCT	Randomized Controlled Trial
REML	Restricted Maximum Likelihood
SAE	Serious Adverse Event
SARS-CoV-2	Severe Acute Respiratory Syndrome Coronavirus 2
S/CO	Signal-to-Cutoff
sVNT	Surrogate Virus Neutralization Test
Th1	T-helper Type 1
WHO	World Health Organization
