# Supplementary material for: Seroconversion Rates Following COVID-19 Vaccination in People Living with HIV: A Systematic Review and Meta-Analysis
Source: Vaccines (Basel). 2026 Jul 23;14(8):648. doi: 10.3390/vaccines14080648 (PMC13517576; doi:10.3390/vaccines14080648)
Supplement: Supplementary file 1 [file vaccines-14-00648-s001.zip › vaccines-4443498-supplementary.pdf]

**Electronic Supplementary Table S1. Search strategy in PubMed.**

| |
|---|
| (COVID-19 vaccine[Title/Abstract] OR COVID vaccine[Title/Abstract] OR Coronavirus vaccine[Title/Abstract] OR mRNA vaccine[Title/Abstract]) AND (random*[Title/Abstract] OR randomized[Title/Abstract] OR randomised[Title/Abstract]) AND (immun*[Title/Abstract] OR immunocompromised[Title/Abstract] OR immunosuppressed[Title/Abstract] OR steroids[Title/Abstract] OR chemotherapy[Title/Abstract] OR transplant[Title/Abstract] OR immunosuppressive[Title/Abstract] OR neoplasm[Title/Abstract] OR cancer[Title/Abstract] OR infection[Title/Abstract] OR autoimmune[Title/Abstract] OR malignancy[Title/Abstract] OR rheumatic[Title/Abstract] OR systemic lupus erythematosus[Title/Abstract] OR HSCT[Title/Abstract] OR CAR-T[Title/Abstract] OR transplantation[Title/Abstract] OR immunotherapy[Title/Abstract] OR tumors[Title/Abstract] OR leukemia[Title/Abstract] OR lymphoma[Title/Abstract] OR inflammatory[Title/Abstract] OR rheumatoid arthritis[Title/Abstract] OR psoriasis[Title/Abstract] OR myasthenia gravis[Title/Abstract] OR HIV[Title/Abstract] OR AIDS[Title/Abstract] OR connective tissue disorder[Title/Abstract] OR neutropenia[Title/Abstract] OR splenectomy[Title/Abstract] OR liver[Title/Abstract] OR kidney[Title/Abstract] OR corticosteroids[Title/Abstract] OR multiple sclerosis[Title/Abstract] OR sarcoidosis[Title/Abstract] OR neuromyelitis optica[Title/Abstract] OR transplantation[Title/Abstract]) |

**Electronic Supplementary Table S2. Summary of Demographic Characteristics of Included Studies.**

| Study (Author, Year); Country | Study Design | Population (N); median age, gender distribution | HIV subgroup | Comorbid disorders | Vaccine(s) Administered (Doses) | On ART | Details of ART medicines | Last follow-up Period (days) - Last day of follow up. |
|---|---|---|---|---|---|---|---|---|
| Antinori 2022 [17] | Prospective observational immunogenicity sub study | PCDR (CD4 <200): 32 patients, 57 years; 25.0% female | PCDR: <200 cells/$mm^3$ (n=32) | Cardiovascular, neurologic, renal, diabetes, COPD/asthma, liver cirrhosis. | BNT162b2 or mRNA-1273. 2-doses | Yes, 100% | NR | 1 month after the second dose. |
| | | ICDR (200-500):56 patients, 54 years; 16.1% female | ICDR: 200-500 cells/$mm^3$ (n=56) | | | | NR | |
| | | HCDR (>500): 78 patients, 54 years; 12.8% female | HCDR: >500 cells/$mm^3$ (n=78) | | | | NR | |
| Ao 2022 [18] | Prospective observational study | 139 patients; median 55 years; 64.0% male / 36.0% female | CD4 <200: 18 (13%); CD4 200-500: 74 (53.2%); CD4 >500: 47 (33.8%)<br>HIV viral load <20 copies/mL: 109 (78.4%) | NR | BBIBP-CorV, CoronaVac, or mixed. 2 doses | Yes, 100% | NNRTI + two NRTIs<br>Boosted PI + one NRTI<br>Boosted PI + two NRTIs | 21-105 days post-vaccination and longitudinal: up to 6 months (180 days) |

| | | | | | | | | |
|---|---|---|---|---|---|---|---|---|
| Balcells 2022 [19] | Prospective cohort | 55 patients, Mean age 46.8 years, Female sex: 2 (3.6%) | PLHIV on ART with CD4 ≤500/μL and HIV viral load <200 copies/mL | Hypertension Diabetes Asthma/COPD | CoronaVac, 2 doses. | Yes, 100% | Not detailed | Blood sample collected 8-12 weeks after 2nd dose. |
| Basso 2022 [20] | Prospective cohort | 184 patients, median age 55 years, 82.6% male | HIV-1 positive adults on successful ART | NR | BNT162b2. 3 doses | Yes. 100% | Not detailed | 6 months after 3rd dose |
| Bergman 2021 [21] | Prospective, open-label, non-randomized clinical trial | 90 patients, Age<65years: 79%, 60% men. (overall) | CD4 > 300 cells/μL (n=60); CD4 < 300 cells/μL (n=30) at baseline. | NR | BNT162b2, 2 doses. | Yes, 100% | NR | Day 35 after first dose. |
| Ogbe 2022 [22] | Open-label, non-randomized substudy of phase II/III COV002 trial | 54 patients; median 42.5 years, 100% male | Well-controlled HIV on ART; undetectable viral load (<50 HIV RNA copies/mL); CD4+ T cells | NR | ChAdOx1 nCoV-19 (AZD1222), 2 doses | Yes, 100% | NR | 182 days |

| | | | | | | | | |
|---|---|---|---|---|---|---|---|---|
| | | | > 350 cells/μL | | | | | |
| Chan 2022 [23] | Prospective cohort | 122 patients; median age 49; 86% male | PLHIV, all receiving ART | NR | CoronaVac, 3 doses | Yes, 100% | NR | Within 90 days after 3rd dose |
| Cheng 2022 [25] | Retrospective substudy within a Phase 2, prospective, double-blinded, multi-centre trial | 57 patients; mean age 38.6 years; 94.7% male | People living with HIV (PWH) on stable ART, CD4+ >350 cells/mm³, HIV viral load <1000 copies/mL | Not detailed | MVC-COV1901, 2 doses | Yes, 100% | NR | Follow-up up to 6 months after second dose. |
| Cossu 2023 [24] | Prospective cohort | 53 patients; median 55 years, 75.5% male | All PWH on ART; 59.6% had history of AIDS; 98.1% virologically suppressed at first dose | Dyslipidemia<br>CVD<br>Diabetes<br>Cirrhosis<br>COPD<br>Obesity<br>Solid Neoplasms<br>Hematological neoplasms | BNT162b2. 2 doses | Yes (100%) | NR | 7 months later |
| Costinuik 2022 [26] | Multi-center prospective observational cohort | 294 patients; median 54.4 years; 77% male | PWH. 20% history of AIDS-defining illness | Obesity<br>Dyslipidemia<br>Hypertension | BNT162b2 or mRNA-1273 or ChAdOx1 or Ad26.COV2. S Combinations (mRNA–mRNA | 97.6% | NRTI-based regimen or NNRTI-based regimen or INSTI-based | 1 month post 3rd dose. |

| | | | | | | | | |
|---|---|---|---|---|---|---|---|---|
| | | | | | or ChAdOx1–mRNA or ChAdOx1–ChAdOx1 1 or Ad26.COV2. S) . 3 doses. | | regimen or PI based | |
| Feng 2021 [28] | Open-label, two-arm non-randomized cohort study | 42 patients; mean age 42.74 years; 29M/13F | HIV-1 infected, stable on cART, CD4+ >200 cells/μL | NR | BBIBP-CorV, 2 doses | Yes, 100% | NR | 4 weeks after vaccination |
| Fusco 2023 [27] | Retrospective observational | 235 patients; median age 51 years; 91% male | PLWH on ART | NR | BNT162b2, 3 doses | Yes, 100% | INI-based triple, PI-based triple, NNRTI-based triple, two-drug, other | 64 days after 3rd dose |
| Gianserra 2022 [29] | Prospective observational | 42; median age 53 years; 37 males (88.1%) | PLWH on ART | NR | BNT162b2, 3 doses | Yes, 100% | INSTI-based regimen and dual therapy | 4 weeks after booster dose |
| Gomez 2022 [30] | Multicentre prospective cohort | <200 cells/mm$^3$ (n= 33): 85% male, 56 years<br>200–349 cells/mm$^3$ (n= 61), 80% | <200 cells/mm$^3$ (n= 33), 200–349 cells/mm$^3$ (n= 61), ≥350 cells/mm$^3$ (n= 326). | Cirrhosis, chronic kidney disease, immunosuppressive therapy | BNT162b2 or mRNA-1273 or ChAdOx1 nCoV-19. 2 doses or Ad26.COV2.S. 1 dose | Yes, 100% | NR | Blood drawn 4–8 weeks after last vaccine dose |

| | | | | | | | | |
|---|---|---|---|---|---|---|---|---|
| | | male, median 56 years | | | | | | |
| | | ≥350 cells/mm³ (n= 326), 82% male, 55 years | | | | | | |
| Gomez 2023 [31] | Case-control study nested in a prospective cohort | CD4 <200: 18 patients, 89% male, median 56 years. | PLWH with CD4 <200 cells/mm³ (n=18) vs. CD4 ≥200 (n=36) | NR | BNT162b2 or mRNA-1273 or ChAdOx1 nCoV-19. or Ad26.COV2.S. (primary series). BNT162b2 or mRNA-1273 (booster dose). 3 doses | 52/54 (96%) | NR | 4–8 weeks after booster dose |
| | | CD4 ≥200: 36 patients, 89% male, median 58 years. | | NR | | | | |
| Griessbach 2023 [32] | Randomized controlled trial extension | 283 patients, participants; median age 54 years; 75.0% male | 283/312 (90.7%) PWH | NR | mRNA-1273 or BNT162b2. 3 doses | NR but implied | NR | 8 weeks (±2 weeks) after third vaccination |
| Haidar 2022 [33] | Prospective observational cohort | 94 patients. Mean 57.4 years. Male: 89.4% | All PWH | Cardiac disease Pulmonary disease, Diabetes, CKD, | mRNA-1273 or BNT162b2 or ChAdOx1 nCoV-19. 2 dose or | 100% (94/94) | NR | Median 67 days post-vaccination |

| | | | | | | | | |
|---|---|---|---|---|---|---|---|---|
| | | | | Dyslipidemia, Liver disease, Obesity Cerebrovascular disease | Ad26.COV2.S. 1 dose | | | |
| Han 2022 [35] | Non-interventional (observational) | 47 patients; median age 34 years; 95.7% male | All had ART; 93.6% undetectable viral load | Syphilis Diabetes Cardiovascular disease | Sinovac CoronaVac or Sinopharm; 2 doses | Yes, 100% | 2 NRTIs + I NNRTI<br>2 NRTIs + I INSTI<br>2 NRTIs + IPIS | Interval between detection and complete vaccination, 59 days |
| Hassold 2023 [34] | Prospective cohort | 23 patients; median age 56 years; 70% male | PLWH with low/non-response to primary 2-dose vaccination | Hypertension, Diabetes, Respiratory Disease | BNT162b2/mRNA-1273/ChAdOx1-nCoV19 (primary series), BNT162b2/mRNA-1273 (booster). 3 doses | Yes, 100% | NR | Median 2.4 months after booster dose |
| Heftdal 2021 [36] | Prospective observational cohort | 269 patients; Median 56 years; 90% male | Well-treated PWH (99.6% on ART, 96.7% with HIV RNA <50 copies/mL). | NR | BNT162b2. 2 doses | 99.6% on ART | NR | 2 months after first dose (approx. 5-7 weeks after second dose, given a 3-week interval) |

| | | | | | | | | |
|---|---|---|---|---|---|---|---|---|
| Hensley 2022 [43] | Prospective nationwide observational cohort study | 984 patients, age categories: 18–55 years: 674 (68.5%) 56–65 years: 157 (16.0%) > 65years: 153 (15.6%), 85.2% male | Adults with confirmed HIV infection. | NR | BNT162b2, mRNA-1273, ChAdOx1-S, or Ad26.COV2. S. 2 doses | 99.0% were on ART | NR | Blood draw 4-6 weeks after completed vaccination schedule |
| Bociaga -Jasik 2023 [37] | Observational study | 121 patients; mean age 40.3; 115 males | PLWH. 15.7% had prior AIDS diagnosis. 92.56% had HIV-RNA <50 copies/mL | Hypertension, CVD, Diabetes Obesity Chronic Hepatitis Liver cirrhosis CKD Alcohol abuse | BNT162b2 or mRNA-1273 or ChAdOx1 nCoV-19. 2 doses Ad26.COV2.S. 1 dose | Yes, 100% | Integrase inhibitor-based 2-drug regimen Protease inhibitor-based NNRTIs | Mean 115.1 ± 44.2 days |
| Oyaert 2022 [38] | Observational prospective | 27 patients; median age 47 years; Male/Female: 19/8 | PLWH with CD4 count <350/µL at inclusion | NR | BNT162b2, 2 doses. | Not mentioned | NR | 3 months (T3) after second dose |

| | | | | | | | | |
|---|---|---|---|---|---|---|---|---|
| Jongkees 2023 [39] | Nested single-arm interventional trial | 66 patients; Median age 63 years; 86% male | PWH with hyporesponse (≤300 BAU/mL) | NR | ChAdOx1-S or BNT162b2, 2 doses or Ad26.COV2.S, 1 dose. (primary series) mRNA-1273. (Additional dose) | 97% on antiretroviral | NR | 28 days post-additional vaccination |
| Lamacchia 2022 [40] | Longitudinal prospective study | 8 PLWH; mean age 58.5 gender distribution not specified | PLWH | Cardiovascular disease, major depressive disorder, chronic hepatitis C, severe obesity, Allogenic HSCT in AML | BNT162b2; 4 doses | Yes, 100% | NR | 2 months post-fourth dose |
| Levy 2021 [45] | Prospective open study | 143 patients; mean age 49.8 years; 91.6% male. | PLWH on ART. 95% had undetectable viral load. | Hypertension, Diabetes, Dyslipidemia, IHD, COPD, kidney/liver disease, malignancies, kidney transplants. | BNT162b2. 2 doses | Yes, 100% | Integrase inhibitor-based therapy or two-drug regimen. | Median 18 days after second dose for immunogenicity. |
| Lombardi 2022 [46] | Prospective cohort study | 71 PLWH; mean age 47 | Stable on ART, median. HIV viral | NR | mRNA-1273, 2 doses | Yes, 100% | NRTIs, InSTIs , NNRTIs, PIs | 28 days after second dose |

| | | | | | | | | |
|---|---|---|---|---|---|---|---|---|
| | | years; 84.5% male | load <50 copies/mL | | | | | |
| Lv 2021 [42] | Prospective cohort study | 24 patients; median 44.0 years; 50% male | People living with HIV (PLWH) | None reported | CoronaVac or BIBBP-CorV; 2 doses | NR | NR | 40 days after second injection |
| Madhi 2022 [47] | Randomised, observer-blinded, multicentre, placebo-controlled phase 2A/2B trial | 122 patients; median age 38 years; 30.3% male. | PLWH on stable ART, HIV-1 viral load <1000 copies/mL | Hypertension | NVX-CoV2373; Novavax. 2 doses | Yes, 100% | NR | Immunogenicity assessed at day 35. |
| Mauas 2023 [48] | Prospective observational cohort | 595 patients; mean age 46.1 years; 70.4% male | $CD4^+$ T cells 659 cells/µl | Obesity Diabetes Hypertension Chronic pulmonary disease Chronic heart disease Chronic renal disease Oncological disease | Sputnik V or AstraZeneca or Sinopharm. 2 doses | NR | NR | Antibody measured between days 28–60 after second dose. |
| Milano 2022 [49] | Retrospective observational | 697 patients; median age 53 years; 74.7% male | PLWH, mostly stable on ART | Dyslipidemia Hypertension Diabetes | BNT162b2, 2 doses | 99.8% on ART | NR | 3 months after complete vaccination |

| | | | | | | | | |
|---|---|---|---|---|---|---|---|---|
| | monocentric study | | | Cardiac and/or vascular disease<br>COBD | | | | |
| Nault 2022 [50] | Prospective cohort | 106 patients, age and gender not provided | Stratified by CD4 count: <250, 250–500, >500 cells/mm³ | NR | mRNA-1273. 1 dose | Yes, 100% | NR | 3–4 weeks after first dose |
| Netto 2022 [51] | Prospective cohort study | 215 patients; median 54 years; 60% male | Stratified by CD4 count:<br>CD4 <500 cells/μL: 64 patients<br>CD4 ≥500 cells/μL: 151 patients<br>Viral suppression (<50 copies/mL): 191 (89%) | Smoking<br>Hypertension<br>Diabetes<br>Cardiopathy<br>Dyslipidaemia<br>COPD<br>Asthma<br>Chronic kidney disease<br>Chronic liver disease<br>Neoplasia<br>Previous stroke<br>Active tuberculosis | CoronaVac, 2 doses. | 214/215 (99.5%) | tenofovir + lamivudine + dolutegravir<br>or<br>tenofovir + lamivudine + efavirenz | Day 69 (6 weeks after second dose) |

| | | | | | | | | |
|---|---|---|---|---|---|---|---|---|
| Noe 2022 [52] | Retrospective, non-interventional cohort study | 665 patients; median age 53 years; 84.2% male | PLWH, mostly virologically suppressed (93.5% with HIV RNA <50 copies/mL). | NR | 2-dose mRNA (Comirnaty/Moderna), 2-dose AstraZeneca, 1-dose Janssen, or heterologous AstraZeneca + mRNA vaccination.2 doses | NR | NR | Median time between last vaccine dose and antibody measurement: 49 days (IQR 35-63). Maximum follow-up for antibody kinetics: 12 weeks post-vaccination. |
| Sisteré-Oró 2022 [53] | Observational study | 10 HIV; median 49 years; 70% male | PLWH | NR | BNT162b2. 2 doses | Yes | NR | 3 weeks after 2nd dose |
| Portillo 2022 [54] | Single-center observational study | 131 PLWH; median age 54 years; 70.2% male | PLWH on effective ART | NR | mRNA-1273 or BNT162b2. 2 doses | Yes, 100% | NR | 6 months after first dose |
| Pourcher 2022 [55] | Multicenter cohort study | 97 PWH; median age 48 years; 87.6% male | CD4+ <500 cells/µl, viral load <50 copies/ml on stable ART ≥3 months | Obesity Hypertension COPD Diabetes Allergy | BNT162b2. 2 doses | Yes. 100% | Integrase inhibitors Tritherapy Bitherapy | 1 month after second dose |

| | | | | | | | | |
|---|---|---|---|---|---|---|---|---|
| Rahav 2021 [56] | Prospective cohort | 156 patients; median age 49 87.8% male | HIV on ART | NR | BNT162b2, 2 doses | Yes, 100% | Mainly integrase inhibitor-based regimens | Serology drawn 2–4 weeks post dose 2 |
| Ruddy 2021 [57] | Prospective observational cohort | 14 patients; median age 62 years, 93% male | PWH on ART | NR | BNT162b2 or mRNA-1273. 2 doses | Yes, 100% | NR | Median 29 days after the second dose |
| Schmidt 2022 [44] | Prospective cohort | 82 HIV+, 55 years, female/male ratio: 16/34 | HIV-1-infected on ART with viral load <100 copies/mL | NR | BNT162b2, 2 doses. | Yes, 100% | NR | 155 days post-second dose. |
| Speich 2022 [41] | Open-label, noninferiority randomized controlled trial | 341 PLWH; median age 53 years; 75.8% male | PLWH | History of cardiovascular disease or metabolic syndrome | mRNA-1273 or BNT162b2. 2 doses | Yes, 100% | NR | 12 weeks after first vaccination |
| Spinelli 2022 [58] | Matched case-control observational study | BNT162b2: 75 PLWH; median age 59; 13 women in matched groups | PLWH | NR | mRNA-1273 or BNT162b2. 2 doses | NR but implied | NR | Median 35 days post-second dose |

| | | | | | | | | |
|---|---|---|---|---|---|---|---|---|
| | | mRNA-1273: 25 PLWH; median age 59; 13 women in matched groups | | | | | | |
| Tortellini 2022 [59] | Prospective cohort | 37 patients, (median 61 years; 26 M / 11 F) | PLWH | HCV Ab+: 9/37 (24%) Smokers: 13/37 (35%) | BNT162b2, 3 doses | Yes, 100% | NR | 6 months after (T2) the third dose. |
| Tuan 2021 [60] | Longitudinal cohort study | 78 patients, 82.1% were aged <65 years, 65.4% male | PLWH | Cancer or other immunosuppressive condition, cardiovascular disease, lung disease, diabetes, advanced liver disease, chronic kidney disease | BNT162b2, 2 doses | Yes. 100% | INSTI or NNRTI or Protease Inhibitor | 2-3 weeks after second dose |
| Verburgh 2023 [61] | Prospective cohort study | 195 patients: Median 63.3 years; 94.4% male at birth | Middle-aged and older PWH | Not detailed | BNT162b2, mRNA-1273, ChAdOx1, Ad26.COV2. S or ChAdOx1+BNT162b2. 2 doses or 1 dose | Yes, 100% | NR | 4 to 13 weeks after the last vaccine dose |

| | | | | | | | | |
|---|---|---|---|---|---|---|---|---|
| | | | | | | | | |
| Vergori 2022 [62] | Prospective observational cohort | PCDR (0-200): 44 patients. Median 57 years, 22.7% females | PLWH | Cancer<br>CKD<br>Mild liver disease<br>Hypertension<br>Severe liver disease<br>Autoimmune disease | 3 doses of mRNA-1273 **or** 2 doses of mRNA-1273 + 3rd dose of BNT162b2 **or** 2 doses of BNT162b2 + 3rd dose of mRNA-1273 **or** 3 doses of BNT162b2 **or** 2 doses of ChAdOx nCoV-19 + 3rd dose of mRNA-1273 **or** 1 dose of Ad26.COV2.S + 1 dose of mRNA-1273 | Yes, 100% | NR | Median time between the third dose and response measurement was 16 days |
| | | ICDR (201-500): 96, median 55 years, 17.7% female | | | | Yes, 100% | NR | |
| | | HCDR (>500 ): 76 patients, Median 52 age, 15.8 % female | | | | Yes, 100% | NR | |
| Wong 2022 [63] | Prospective longitudinal observational study | BNT162b2: 423 patients, median 41 years, 93% male | Adults with HIV in care; median years since HIV diagnosis 6.45 | NR | BNT162b2.2 doses | NR | NR | 6 months after second dose |
| | | CoronaVac / Sinovac: 184 patients, | | | CoronaVac. 2 doses | NR | NR | |

| | | | | | | | | |
|---|---|---|---|---|---|---|---|---|
| | | median 50 years, 86 % male | | | | | | |
| Wu 2021 [66] | Prospective cohort study | 259 patients; Age 18–59 (median not reported); Male: 240 (92.7%), | PLWH | NR | Inactivated COVID-19 vaccine (Sinopharm). 2 doses | 99.2% on ART | NNRTIs INSTIs PIs | 7 days after each dose |
| Wu 2023 [72] | Prospective cohort study | 138 PLWH, median age 38 years, 88.4% male | PLWH (CD4 stratified: <200, 200–500, ≥500/µL) | Hypertension, diabetes, Hyperlipidemia, Cancer, chronic cardiovascular and lung, liver, or kidney diseases. | Inactivated COVID-19 vaccine (Sinopharm). 2 doses | 91.3% on ART | NNRTIs INSTIs PIs | 3rd month after second dose |
| Zeng 2022 [65] | Prospective longitudinal cohort | BBIBP-CorV: 65 patients, 119 (90.2%) male, median 32 years (overall) | PLWH aged 18-59, on routine follow-up | NR | BBIBP-CorV or CoronaVac; 2 doses | Majority of the PLWH (87.9%) had received stable ART for more than 6 months | NR | 180 days after second dose |

| | | | | | | | | |
|---|---|---|---|---|---|---|---|---|
| | | CoronaVac: 67 patients, 119 (90.2%) male, median 32 years (overall) | | NR | | | NR | |
| Zou 2022 [68] | Longitudinal cohort | 46 patients; 36 years; 87% male | Majority with CD4 >350/µL, viral suppression (89% with HIV RNA <50 copies/mL) | NR | Sinopharm, WIBP-CorV, 2 doses | Yes, 100% | NNRTI-based<br>Integrase inhibitor-based<br>PIs-based | Day 190 |
| Aledo 2022 [64] | Prospective cohort study | 100 patients; Mean age 44 years; 75% male | Well-controlled PWH (98% HIV RNA <50 copies/mL; 96% CD4+ >200/µl) | HBV infection<br>HCV infection | mRNA-1273 or BNT162b2; 2 doses | Yes, 100% | INSTI+2NRTIs or INSTI+1NRTI, PI-based, NNRTI+2 NRTIs | 4 weeks after second dose |
| Chittrakarn 2024 [67] | Prospective cohort study | CD4 ≤ 200 cells/µL: 15 patients; Median age 47 years, 67% males | CD4 (≤200 cells/µL): n=15; or CD4 (>200 cells/µL): n=15 | Mild hemophilia A, Metabolic disease, Panic disorder. | CoronaVac (Sinovac) + Vaxzevria (AstraZeneca). 2 doses | Yes, 100% | NNRTIs<br>PIs<br>INSTIs | Day 84 after complete vaccination |

| | | | | | | | | |
|---|---|---|---|---|---|---|---|---|
| | | CD4 > 200 cells/µL: 15 patients; Median age 47 years, 53% males | | | | | | |
| Li 2023 [69] | Longitudinal cohort study | HIV-infected (Nadir ≤350): 59 patients, median 35 years, 95% males (overall) | Stratified by nadir CD4+ T cell count: - ≤350 cells/mm³ - >350 cells/mm³ | Hepatitis B Hepatitis C STD | CoronaVac (SinoVac) or Covilo (Sinopharm). 3 doses | Yes, 100% | 2 NRTIs + 1 NNRTI (free drugs) Protease inhibitors + 2 NRTIs Integrase inhibitors + 1-2 NRTIs | 14 days after 3rd dose |
| | | HIV-infected (Nadir >350): 42 patients, median 35 years, 95% male (overall) | | | | | | |
| Loubet 2022 [70] | Prospective, observational cohort study | 897 patients, gender and age NR | PLWHIV | NR | BNT162b2 or mRNA-1273 or mixed. 2 doses | NR | NR | 1 month after dose 2 and after dose 3 |
| Lv 2023 [71] | Clinical trial (open-label, non-randomized) | 34 PLWH, median age 43.5 years, 50% male | PLWH | NR | CoronaVac or BIBP-CorV, 3 doses | Yes, 100% | TDF+3TC+ EFV or TDF+3TC+ LPV/r or AZT+3TC+ LPV/r or AZT+3TC+ | Approximately 18 days post-third dose |

| | | | | | | | | |
|---|---|---|---|---|---|---|---|---|
| | | | | | | | NVP or AZT+3TC+ EFV or others | |
| Lu 2023 [73] | Longitudinal observational study | 100 patients; median age 57 years; 63% male. | < 200 cells/μL: n = 20 (20% of total) 200-500 cells/μL: n = 55 (55%) > 500 cells/μL: n = 25 (25%) | NR | BBIBP-CorV or CoronaVac. 3 doses | Yes, 100% | NR | 6 months after booster vaccination (T6) |
| Morrocchi 2022 [74] | Prospective observational | 28 patients; mean age 24.7 years; 50% female | Perinatally HIV-infected adolescents and young adults | NR | BNT162b2, 2 doses | Yes, 100% | INI+NRTI, INI+PI, INI+2NRTIs, 2NRTIs+PI, others | 180 days later |

| | | | | | | | | |
|---|---|---|---|---|---|---|---|---|
| Wang 2024 [75] | Multicenter prospective cohort study | 228 patients; median age 34 ; 96.05% male | CD4 ≥350: 191 (83.77%)<br>CD4 <350: 37 (16.23%) | Liver disease<br>Hypertension<br>Hyperlipoidemi<br>Allergic disease<br>Diabetes<br>Cardiovascular disease | CoronaVac, 3 doses | 91.23% were on ART | TDF+3TC+EFV<br>TDF+3TC+NVP<br>TDF+3TC+LPV/r<br>AZT+3TC+LPV/r<br>AZT+3TC+NVP<br>AZT+3TC+EFV | 6 months post-booster dose |
| Yu 2023 [76] | Prospective cohort | 236 patients, gender and age not mentioned | People living with HIV-1 (PLWH) | Diabetes, hypertension, cancer, cardiovascular diseases, hepatitis, renal insufficiency | Inactivated SARS-CoV-2 vaccine (CoronaVac or others), 3 doses | Yes (n=77) and No (n=29) | Not detailed | NR |

**Electronic Supplementary Table S3. Summary of Serological Characteristics of Included Studies.**

| Study (Author, Year) | Population Analyzed (N) | Assay type | Baseline COVID-19 status | Seroconversion definition | CD4 baseline | CD4 response | Seroconversion Rate n/N (%) * | Geometric Mean/Median Titer [95% CI/IQR] | Vaccine Effectiveness |
|---|---|---|---|---|---|---|---|---|---|
| **Antinori 2022** | **PCDR (CD4 <200): 32 patients** | **Anti-S/RBD IgG (ARCHITECT), microneutralization assay (MNA), IFN-γ release (ELLA)** | **No prior Covid 19** | **Detectable RBD-binding IgG (≥7.1 BAU/mL)** | **PCDR (CD4 <200)** | **NR** | **26/30 (86.7%)** | **NR** | **NR** |
| | **ICDR (200-500): 56 patients** | | | | **ICDR (200-500)** | **NR** | **53/53 (100%)** | **NR** | **NR** |
| | **HCDR (>500): 78 patients** | | | | **HCDR (>500)** | **NR** | **76/77 (98.7%)** | **NR** | **NR** |

| | | | | | | | | | |
|---|---|---|---|---|---|---|---|---|---|
| **Ao 2022** | **139 patients** | **Indirect ELISA (anti-RBD-IgG, anti-spike-IgG); Flow cytometry (RBD-specific memory B cells)** | **No prior Covid 19** | **Anti-RBD-IgG: OD value ≥2.1× mean absorbance of negative controls at 1:50 dilution;<br><br>Anti-spike-IgG: >1.495 AU/mL** | **Not detailed** | **NR** | **121/139 (87.1%)** | **134.2 [95% CI: 114.0 158.0]** | **NR** |
| **Balcells 2022** | **55 patients** | **Surrogate virus neutralization test (sVNT) (GenScript)<br><br>SARS-CoV-2 QuantiVac ELISA (Euroimmun)** | **No prior Covid 19** | **ELISA ≥ 11 (RU) per milliliter.** | **Mean 358.8 cells/µL** | **NR** | **39 / 55 (70.9%)** | **NR** | **NR** |
| **Basso 2022** | **184 patients** | **DiaSorin Liaison SARS-CoV-2 TrimericS IgG** | **No prior Covid 19** | **>33.8 BAU/mL** | **Median 639 cells/mm³** | **NR** | **183/184 (99.5%)** | **NR** | **NR** |
| **Bergman 2021** | **CD4 > 300 cells/ µL group: 54 patients** | **Elecsys Anti-SARS-CoV-2 S (Roche)** | **No prior Covid 19** | **≥0.80 U/mL** | **>300 cells/µL** | **NR** | **54/54 (100%)** | **NR** | **NR** |

| | | | | | | | | | |
|---|---|---|---|---|---|---|---|---|---|
| | CD4 < 300 cells/ µL: 25 patients | | | | ≤300 cells/µL | | 24/25 (96%) | | |
| Ogbe 2022 | 54 patients | Serology (IgG ELISA, MSD), Neutralization (FRNT), ACE-2 inhibition, IFN-γ ELISpot, AIM assay, T cell proliferation | Negative | Anti-spike IgG >13 EU (ELISA) or >1160 AU/mL (S); anti-RBD >1169 AU/mL) | Median 694 cells/µL | NR | Anti-Spike IgG: 35/42 (83.3%) Anti-RBD IgG: 27/42 (64.2%) | NR | NR |
| Chan 2022 | 122 patients | Surrogate Virus Neutralization Test (sVNT) | NR | sVNT ≥ 30% inhibition | Median 564.5 cells/µL | NR | 111/122 (91%) | NR | NR |
| Cheng 2022 | 57 patients | Live virus neutralization assay | NR | ≥4-fold rise from baseline | CD4+ T cell count >350 cells/mm³ | NR | 57/57 (100%) | HIV+: 137.7 IU/mL (110.7–171.3) | NR |
| Cossu 2023 | 53 patients | LIAISON SARS-CoV-2 TrimericS IgG assay (DiaSorin) for anti-S IgG; Elecsys anti-N test (Roche) | No prior Covid 19 | Anti-S IgG ≥33.8 BAU/mL | Median CD4 570/µL | NR | 50/53 (94.3%) | NR | NR |

| | | | | | | | | | |
|---|---|---|---|---|---|---|---|---|---|
| **Costinuik 2022** | **294 patients** | **High-throughput chemiluminescent ELISA for anti-S, anti-RBD, anti-N IgG** | **No Prior Covid 19** | **Co-positivity for anti-S and anti-RBD IgG (signal/cutoff ≥1.0)** | **Median 650 cells/µl** | **NR** | **100% (122/122)** | **NR** | **NR** |
| **Feng 2021** | **42 patients** | **Anti-spike IgG ELISA and surrogate neutralization assays.** | **No prior Covid 19** | **≥3-fold increase from baseline** | **Mean 659 ± 222 cells/µL** | **Mean 477 ± 151 cells/µL** | **24/42 (57.1%)** | **NR** | **NR** |
| **Fusco 2023** | **235 patients** | **Chemiluminescence (SARS-CoV-2 anti-spike IgG S1/S2/RBD, Diasorin); Antinucleocapsid: Roche electrochemiluminescence** | **No prior COVID-19** | **Anti-spike >33.8 BAU/mL** | **Median 663 cells/µL** | **Median 707 cells/µL** | **63/64 (98%)** | **NR** | **19 breakthrough infections after second dose + 15 after 3rd dose** |
| **Gianserra 2022** | **42 patients** | **LIAISON SARS-CoV-2 S1/S2 IgG test (DiaSorin) – chemiluminescent immunoassay (CLIA)** | **No prior Covid 19 patients** | **≥15 AU/mL** | **Median 687 cells/mm³** | **732 cells/mm³ at T3** | **42/42 (100%)** | **NR** | **None had breakthrough infections** |

| | | | | | | | | | |
|---|---|---|---|---|---|---|---|---|---|
| **Gomez 2022** | **<200 cells/mm³, 33 patients** | **Anti-S IgG (LIAISON); Neutralizing IgG (VIRCELL)** | **No prior Covid 19** | **Anti-S ≥33.8 BAU/mL** | **<200, 200–349, ≥350 cells/mm³** | **NR** | **CD4 <200: 21/33 (64%)** | **NR** | **NR** |
| | **200–349 cells/mm³, 66 patients** | | | | | | **CD4 200–349: 55/61 (90%)** | | |
| | **≥350 cells/mm³ (n =326)** | | | | | | **CD4 ≥350: 308/326 (95%)** | | |
| **Gomez 2023** | **CD4 <200: 18 patients** | **LIAISON® SARS CoV-2 TrimericS IgG assay (DiaSorin)** | **No prior Covid 19** | **Anti-S levels ≥33.8 BAU/mL** | **CD4 <200 cells/mm³ or CD4 ≥200** | **NR** | **CD4 <200: 15/18 (83%)** | **NR** | **NR** |
| | **CD4 ≥200: 36 patients** | | | | | | **CD4 ≥200: 36/36 (100%)** | | |

| | | | | | | | | | |
|---|---|---|---|---|---|---|---|---|---|
| Griessbach 2023 | 283 patients | Elecsys Anti-SARS-CoV-2 S (Roche) | NR | ≥100 units/mL | >350 cells/µL: 262/312 (84.0%) | Not reported | 276/277 (99.6%) | Not reported | NR |
| Haidar 2022 | CD4 >200 cells/µL: 82 patients | Beckman Coulter SARS-CoV-2 IgG (anti-RBD); pseudovirus neutralization assay (subset) | Excluded if known prior COVID-19 | S/CO ratio ≥1.00 | CD4 >200 cells/µL: 82/94 (87.2%) | NR | 86.6% (71/82) | NR | 2 breakthrough infections |
| | CD4 <200 cells/µL: 12 patients | | | | CD4 <200 cells/µL: 12/94 (12.8%) | | 4/12 (33.3%) | NR | |
| Han 2022 | 47 patients | Magnetic particle chemiluminescence immunoassay | No prior Covid 19 | IgG ≥1.2 S/CO | Mean 433 cells/µL | Not detailed | 7/10 (70%) | NR | NR |
| Hassold 2023 | 23 PLWH | Architect SARS-CoV-2 IgG II Quant (Abbott) – anti-Spike IgG | No prior Covid 19 | ≥260 BAU/mL | Median 386 cells/µL | Median 556 cells/µL | 17/23 (74%) | NR | NR |
| Heftdal 2021 | 269 patients | In-house ELISA for IgG (RBD); In-house pseudo-neutralizing assay (ACE-2/RBD inhibition) | No prior Covid 19 | RBD IgG ≥150 AU/ml AND neutralizing index ≥25% | Median current CD4: 640 cells/mm³ | NR | 263/269 (98%) | NR | NR |

| | | | | | | | | | |
|---|---|---|---|---|---|---|---|---|---|
| **Hensley 2022** | **984 patients** | **LIAISON chemiluminescence immunoassay (DiaSorin) for anti-spike (S1) IgG Or ELISpot (IFN-γ) and AIM assay** | **No prior Covid 19** | **≥33.8 BAU/mL** | **Median 710 cells/μL** | **NR** | **1145/1154 (99%)** | **NR** | **NR** |
| **Bociaga-Jasik 2023** | **121 patients** | **Anti-SARS-CoV-2 IgG ELISA (Euroimmun, S1 protein).** | **21 (17.3%) reported prior SARS-CoV-2 infection** | **IgG >30 BAU/mL** | **Mean 878 cells/μL** | **NR** | **95/121 (78.5%).** | **95.7 BAU/mL [Q1-Q3 37.9-324.6]** | **NR** |
| **Oyaert 2022** | **27 patients** | **Anti-SARS-CoV-2 TrimericS IgG (CLIA, Diasorin); QuantiFERON SARS-CoV-2 (IFN-γ release)** | **5/27 (18.5%) had detectable anti-spike antibodies at baseline** | **Anti-S IgG ≥33.8 BAU/mL;** | **Median 254/μL** | **NR** | **25/25 (100%)** | **NR** | **NR** |

| | | | | | | | | | |
|---|---|---|---|---|---|---|---|---|---|
| **Jongkees 2023** | **66 patients** | **DiaSorin Liaison IgG trimeric chemiluminescence immunoassay and Plaque reduction neutralization test (PRNT50) flow cytometry** | **No prior Covid 19** | **>300 BAU/mL** | **Median 650 cells/µL** | **NR** | **64/66 (97%)** | **NR** | **NR** |
| **Lamacchia 2022** | **8 PLWH** | **Chemiluminescent immunoassay/ flow cytometry** | **No prior Covid 19** | **Anti-spike IgG cut-off: 87.8 BAU/mL Anti-RBD IgG cut-off: 130 BAU/mL** | **Mean 548.2 cells/mm³** | **Not detailed** | **8/8 (100%)** | **NR** | **Breakthrough infections: 2 patients** |
| **Levy 2021** | **143 patients** | **ELISA for anti-RBD IgG.** | **No prior Covid 19** | **Anti-RBD IgG titre ≥1.1 defined as positive.** | **Geometric mean 700 cells/µL** | **Geometric mean CD4 decreased to 531 after dose 1 and 634 after dose 2.** | **139/143 (97.2%)** | **5.17 (95% CI 4.84-5.53)** | **NR** |
| **Lombardi 2022** | **71 PLWHIV** | **Anti-spike (anti-S) total Ig (ECLIA); VSV-based pseudovirus neutralization (ND50)** | **9 PLWHIV (12.7%) had prior infection** | **Positive anti-S antibodies at T1b** | **Median CD4+ T cell count 747 cells/µL** | **NR** | **71/71 (100%)** | **NR** | **NR** |

| | | | | | | | | | |
|---|---|---|---|---|---|---|---|---|---|
| Lv 2021 | 24 patients | Competitive ELISA for anti-SARS-CoV-2 neutralizing antibody (inhibition of ACE2-RBD binding) | No prior Covid 19 | Percent inhibition ≥20% | NR | NR | 19/24 (79.17%) | NR | NR |
| Li 2023 | HIV-infected (Nadir ≤350): 59 patients | Chemiluminescent immunoassay (CLIA) - anti-RBD IgG | No prior Covid 19 | S/CO value ≥1.0 | Mean 668.83 ± 226.75 | NR | 30/30 (100%) | NR | NR |
| | HIV-infected (Nadir >350): 42 patients | | | | | NR | 27/27 (100%) | NR | NR |
| Madhi 2022 | 122 patients | Anti-spike IgG ELISA; ACE-2 receptor-binding inhibition; wild-type neutralising antibody. | 35·2% were positive on baseline | ≥4-fold rise in titre from day 0 | Median 729.5 cells/µL | NR | 94/97 (96·9%) | 31210·8 (22 665·4–42 977·9) | NR |

| | | | | | | | | | |
|---|---|---|---|---|---|---|---|---|---|
| Mauas 2023 | 80 patients | ADVIA Centaur SARS-CoV-2 IgG chemiluminescent immunoassay (Siemens) | 8.4% had prior COVID-19 | IgG antibody titers ≥1 U/ml | Mean 649.8 cells/µl | NR | 76/80 (95.0%) | NR | NR |
| Milano 2022 | 694 patients | Chemiluminescent microparticle immunoassay (Abbott) detecting IgG anti-RBD | 1.1% had prior documented SARS-CoV-2 infection | ≥50 AU/ml | CD4+ cells % a median (range): 35 (0−59.2) | NR | 484/491 (98.6%) | NR | NR |
| Nault 2022 | 106 patients | ELISA (anti-RBD IgG) | No prior Covid 19 | Mean of pre-pandemic plasma + 3 SD | CD4 strata: <250, 250-500, >500 cells/mm³. | NR | 100/106 (94.3%) | NR | NR |
| Netto 2022 | 215 patients | Chemiluminescent immunoassay (LIAISON SARS-CoV-2 S1/S2 IgG, DiaSorin)<br><br>virus neutralization test (GenScript) | No prior Covid 19 | ≥15·0 AU/µL | Median CD4: 655 cells/µL | NR | 185/204 (91%) | 22.6 (IQR 10.9-41.2) | NR |

| | | | | | | | | | |
|---|---|---|---|---|---|---|---|---|---|
| **Noe 2022** | **665 PLWH** | **LIAISON SARS-CoV-2 TrimericS IgG assay (Diasorin, Italy)** | **No prior Covid 19** | **≥ 34 BAU/mL** | **Median 708 cells/µL** | **Not detailed** | **97.3% (647/665)** | **NR** | **NR** |
| **Sisteré-Oró 2022** | **10 patients** | **ELISA (anti-Spike IgG), NeutraLISA (RBD neutralization), IFN-γ ELISpot** | **No prior Covid 19** | **IgG endpoint titer >1:2700; Neutralization % inhibition ≥35%** | **8 patients: <200 cells/mm³; 2 patients: 200–349 cells/mm³** | **NR** | **5/10 (50%)** | **NR** | **NR** |
| **Portillo 2022** | **131 PLWH** | **Anti-SARS-CoV-2 S1-receptor binding domain (RBD) antibodies (Roche Elecsys)** | **21.5% (28/130) anti-N positive** | **Anti-RBD >1.1 IU/ml** | **Median 602 cells/µl** | **NR** | **95/95 (100%)** | **1303.4 IU/ml (1075.7-1579.2)** | **NR** |
| **Pourcher 2022** | **97 PWH** | **Anti-RBD IgG (Abbott); Neutralization (GenScript ELISA + VNT for D614G, beta, delta)** | **15.5% anti-N IgG+ at baseline; 23% anti-RBD IgG+ at baseline** | **Anti-RBD IgG ≥7.1 BAU/ml; NAb inhibition ≥30% (ELISA) or titer >1:10 (VNT)** | **Median 401 cells/µl** | **NR** | **87/90 (97%)** | **NR** | **NR** |
| **Rahav 2021** | **156 patients** | **RBD-IgG ELISA; pseudovirus neutralization assay** | **No prior Covid 19** | **RBD-IgG titer ≥1.1** | **Mean CD4+ T cell count: 700 cells/µL** | **Not reported** | **154/156 (98.7%)** | **5.14 (95% CI 4.84–5.46);** | **NR** |

| | | | | | | | | | |
|---|---|---|---|---|---|---|---|---|---|
| **Ruddy 2021** | **14 PWH** | **Roche Elecsys anti-SARS-CoV-2 S EIA** | **None had prior COVID-19** | **≥0.8 U/mL** | **<200: n=2<br>200-349: n=1<br>350-499: n=3<br>≥500: n=8** | **NR** | **14/14 (100%)** | **NR** | **NR** |
| **Schmidt 2022** | **82 patients** | **ELISA: Anti-spike IgG/IgA (Euroimmun) Surrogate Neutralization: ACE2/RBD inhibition (Euroimmun NeutralISA)** | **NR** | **IgG/IgA: Ratio ≥1.1 Neutralization: Inhibition ≥35%** | **Median 634/µL** | **NR** | **50/50 (100%)** | **NR** | **NR** |
| **Speich 2022** | **341 patients** | **Elecsys Anti-SARS-CoV-2 S (Roche); ABCORA 2** | **Prior infection: 9.1% overall** | **Elecsys S: ≥0.8 U/Ml, ABCORA 2: positive response; ABCORA 2 sum S1 >17 for neutralization** | **PLWH stratified by CD4 count** | **NR** | **341/341 (100%)** | **NR** | **NR** |

| | | | | | | | | | |
|---|---|---|---|---|---|---|---|---|---|
| **Spinelli 2022** | **BNT162b2: 75 PLWH** | **sVNT (surrogate virus neutralization test); anti-RBD IgG** | **No prior Covid 19** | **sVNT reciprocal titer ≥ 10** | **Median 511 cells/uL** | **NR** | **54/75 (72%)** | **NR** | **NR** |
| | **mRNA-1273: 25 PLWH** | | | | | | **22/25 (88%)** | **NR** | **NR** |
| **Tortellini 2022** | **37 patients** | **DiaSorin Liaison SARS-CoV-2 TrimericS IgG (CLIA)** | **No prior Covid 19** | **≥33.8 BAU/mL** | **Median 547 cells/µL** | **NR** | **29/31 (94%)** | **NR** | **NR** |
| **Tuan 2021** | **78 patients** | **Healgen COVID-19 anti-Spike IgG/IgM Rapid Test** | **No prior Covid 19** | **Positive anti-Spike IgG** | **CD4 ≥500 cells/µL or CD4 <500 cells/µL** | **NR** | **39/40 (97.5%)** | **NR** | **NR** |

| | | | | | | | | | |
|---|---|---|---|---|---|---|---|---|---|
| **Verburgh 2023** | **195 patients** | **Luminex immunoassay (MFI) Neutralization MTT or N protein ELISA (ID50) ELISA IFN-γ release ELISA; AIM assay** | **Prior infection: 14.4% PWH** | **Anti-S IgG titer ≥ 17.8 MFI** | **Median 640/mm$^3$** | **NR** | **189/195 (96.9%)** | **NR** | **NR** |
| **Vergori 2022** | **PCDR (<200 ): 44 patients** | **Anti-RBD IgG (CMIA), Abbott, Micro-neutralization (MNA90), IFN-γ release (ELISA)** | **No prior Covid 19** | **Anti-RBD: ≥7.1 BAU/mL; nAbs: ≥1:10;** | **PCDR (0-200), ICDR (201-500), HCDR (500+)** | **NR** | **42/44 (95.5%)** | **NR** | **NR** |
| | **ICDR (200-500): 96 patients** | | | | | | **96/96 (100%)** | **NR** | **NR** |
| | **HCDR (>500 ): 76 patients** | | | | | | **76/76 (100%)** | **NR** | **NR** |
| **Wong 2022** | **BNT162b2: 423 patients** | **Surrogate virus neutralization test (sVNT) – based on** | **No prior Covid 19** | **sVNT inhibition >20%** | **Median 262 cells/µL** | **Not reported** | **83 / 88 (94%)** | **NR** | **NR** |

| | | | | | | | | | |
|---|---|---|---|---|---|---|---|---|---|
| | | **antibody-mediated blockage of ACE2-spike RBD interaction (GenScript)** | | | **Median 181 cells/µL** | | **20 / 35 (57%)** | | |
| | **CoronaVac / Sinovac: 184 patients** | | | | | | | | |
| **Wu 2021** | **259 patients** | **NR** | **NR** | **NR** | **NR** | **NR** | **NR** | **NR** | **NR** |
| **Wu 2023** | **138 PLWH** | **Surrogate virus neutralization test for nAbs; ELISA for IgM/IgG** | **No prior Covid 19** | **≥0.18 EU/mL** | **Median 495 cells/µL** | **Not reported** | **62/138 (44.9%)** | **0.10 (0.074–0.13)** | **NR** |
| **Zeng 2022** | **BBIBP-CorV: 65 patients** | **Chemiluminescence for S-RBD-IgG** | **No prior Covid 19** | **S/CO value ≥ 1.0** | **Median 505 cells/µL for all PLWH** | **NR** | **53/65 (81.5%)** | **NR** | **NR** |
| | **CoronaVac: 67 patients** | | | | | | **58/67 (86.6%)** | **NR** | **NR** |

| | | | | | | | | | |
|---|---|---|---|---|---|---|---|---|---|
| **Zou 2022** | **46 patients** | **Surrogate virus neutralization test (nAbs); ELISA** | **No prior Covid 19** | **≥0.18 EU/mL** | **Median 523 cells/µl** | **Not reported** | **8/46 (18%)** | **NR** | **NR** |
| **Aledo 2022** | **100 patients** | **LIAISON SARS-CoV-2 TrimericS IgG (CLIA)** | **No prior Covid 19** | **≥33.8 BAU/ml** | **Mean 785 ± 407 cells/µl** | **Median change: -17 cells/µl** | **100/100 (100%)** | **NR** | **NR** |
| **Chittrakarn 2024** | **CD4 ≤ 200 cells/µL: 15 patients** | **Elecsys anti-SARS-CoV-2 S cPass surrogate virus neutralization test (sVNT)** | **No prior Covid 19** | **≥68% inhibition** | **Median 139 cells/µL (IQR 129-149.5)** | **NR** | **7/15 (46.7%)** | **NR** | **NR** |
| | **CD4 > 200 cells/µL: 15 patients** | | | | **Median 575 cells/µL (IQR 476.5-681)** | **NR** | **10/15 (66.7%)** | **NR** | |
| **Loubet 2022** | **897 patients** | **Anti-Spike SARS-CoV-2 IgG (ELISA); Anti-SARS-CoV-2-specific neutralizing antibodies: In vitro neutralization assay** | **No prior Covid 19 infection** | **Anti-Spike SARS-CoV-2 IgG antibodies positive (ratio ≥1.1)**<br>**Neutralizing: titre ≥20.** | **NR** | **NR** | **885/897 (98.7%)** | **NR** | **NR** |

| | | | | | | | | | |
|---|---|---|---|---|---|---|---|---|---|
| **Lv 2023** | **34 patients** | **Neutralizing Ab: competitive ELISA; IgG: chemiluminescent microparticle immunoassay** | **No prior COVID-19** | **≥20% inhibition for neutralizing antibody** | **<500: 17 (50%); 500–1000: 16 (47.06%); >1000: 1 (2.94%)** | **NR** | **32/34 (94.11%)** | **NR** | **NR** |
| **Lu 2023** | **100 patients** | **Chemiluminescence immunoassay (Neutralizing Abs - NAbs; anti-RBD-IgG)** | **NR** | **NAbs: >0.15 µg/mL** | **Median 362 cells/µL** | **NR** | **79/100 (79%)** | **NR** | **NR** |
| **Morrocchi 2022** | **28 patients** | **LIAISON SARS-CoV-2 TrimericS-IgG; Elecsys Anti-SARS-CoV-2 S (Roche)** | **No prior Covid 19** | **Positive IgG** | **NR** | **NR** | **Anti-RBD: 100% (27/27) at D28; Anti-trimeric S: 100% (25/25) at D28** | **NR** | **NR** |
| **Wang 2024** | **PLWH with CD4 < 350 (N=37)** | **nAbs (CLIA), S-IgG (CLIA)** | **No prior Covid 19** | **nAbs > 11.5 IU/mL; S-IgG > 20 IU/mL** | **CD4 <350** | **NR** | **S-IgG Antibodies: 142/194 (73.20%)** | **NR** | **NR** |
| | **PLWH with CD4 ≥ 350 (N=191)** | | | | **CD4 ≥350** | **NR** | | | |

| | | | | | | | | | |
|---|---|---|---|---|---|---|---|---|---|
| **Yu 2023** | **PLWH not on ART (n=29)** | **nAb (neutralizing antibody); RBD-IgG ELISA** | **No history of SARS-CoV-2 infection** | **nAb positive** | **<200 (n=9)** | **Not applicable (No ART)** | **1/9 (11%)** | **NR** | **NR** |
| | | | | | **>200 (n=20)** | | **17/20 (85%)** | **NR** | |
| | **PLWH on ART (n=77)** | **nAb; RBD-IgG ELISA** | **No history of SARS-CoV-2 infection** | **nAb positive** | **<200 (n=20)** | **On ART** | **10/20 (50%)** | **NR** | |
| | | | | | **>200 (n=33)** | | **28/33 (85%)** | **NR** | |
| | | | | | **>500 (n=25)** | | **24/25 (96%)** | **NR** | |

**Electronic Supplementary Table S4. Summary of Safety Characteristics of Included Studies.**

| Study (Author, Year) | Population Analyzed (N) | Any Local AE n/N | Any Systemic AE n/N | Serious Adverse Events (SAEs) n/N (%) | SAEs Possibly Related to Vaccine (always unrelated) - if its possibly related. | Key Findings / Notes |
|---|---|---|---|---|---|---|
| Antinori 2022 | PCDR (CD4 <200) | NR | NR | NR | NR | NR |
| | ICDR (200-500) | NR | NR | NR | NR | NR |
| | HCDR (>500) | NR | NR | NR | NR | NR |
| Ao 2022 | 139 patients | Pain 12/139 (8.6%), Swelling 1/139 (0.7%), Redness 1/139 (0.7%), Itch 1/139 (0.7%) | Fatigue 3/139 (2.2%), rash 2/139 (1/4%) | 0/139 (0%) | NR | NR |
| Balcells 2022 | 55 patients | NR | NR | NR | NR | NR |
| Basso 2022 | 184 patients | NR | NR | NR | NR | NR |
| Bergman 2021 | 90 patients | NR | NR | Not detailed | 1 case – vasovagal reaction. | NR |
| Ogbe 2022 | 54 patients | NR | NR | NR | NR | NR |

| | | | | | | |
|---|---|---|---|---|---|---|
| Chan 2022 | 122 patients | NR | NR | NR | NR | NR |
| Cheng 2022 | 58 patients | Pain/Tenderness: 39/58 (67.2%)<br>Induration/Swelling: 1/58 (1.7%) | Malaise/Fatigue: 25/58 (43.1%)<br>Myalgia: 10/58 (17.2%)<br>Headache: 13/58 (22.4%)<br>Diarrhea: 14/58 (24.1%)<br>Nausea/Vomiting: 6 /58(10.3%) | NR | NR | NR |
| Cossu 2023 | 53 patients | NR | NR | NR | NR | NR |
| Costinuik 2022 | 294 patients | Pain at injection: 66% | NR | NR | NR | NR |
| Feng 2021 | 42 patients | NR | NR | NR | NR | NR |
| Fusco 2023 | 235 patients | NR | NR | NR | NR | NR |
| Gianserra 2022 | 42 patients | 10/42 (23.8%) – all mild pain at injection site, resolved within 48h | Headache: 2/42 (4.8%)<br>Fever <24h 3/42 (7.1%)<br>Mild arthromyalgia 1/42 ( 2.4%) | 1/42 (2.4%) – sensorineural hearing loss (SNHL) | Bilateral SNHL | NR |

| | | | | | | |
|---|---|---|---|---|---|---|
| **Gomez 2022** | **<200 cells/mm³ (n=33)** | **NR** | **NR** | **NR** | **NR** | **NR** |
| | **200–349 cells/mm³ (n=61)** | **NR** | **NR** | **NR** | **NR** | **NR** |
| | **≥350 cells/mm³ (n=326)** | **NR** | **NR** | **NR** | **NR** | **NR** |
| **Gomez 2023** | **CD4 <200: 18 patients** | **NR** | **NR** | **NR** | **NR** | **NR** |
| | **CD4 ≥200: 36 patients** | **NR** | **NR** | **NR** | **NR** | **NR** |
| **Griessbach 2023** | **283 patients** | **Not detailed** | **Not detailed** | **Not detailed** | **Not detailed** | **NR** |
| **Haidar 2022** | **CD4 >200 cells/µL: 82 patients** | **NR** | **NR** | **NR** | **NR** | **NR** |
| | **CD4 <200 cells/µL: 12 patients** | **NR** | **NR** | **NR** | **NR** | **NR** |

| | | | | | | |
|---|---|---|---|---|---|---|
| **Han 2022** | **47 patients** | **Injection site pain: 3/47 (6.4%)<br>Swollen: 2/47 (4.2%)<br>Local redness: 2/47 (4.2%)** | **Fatigue: 3/47 (6.4%)<br>Headache: 3/47 (6.4%)<br>Muscle soreness: 3/47 (6.4%)<br>Fever: 2/47 (4.2%)<br>Diarrhea: 1/47 (2.1%)** | **NR** | **NR** | **NR** |
| **Hassold 2023** | **23 patients** | **NR** | **NR** | **NR** | **NR** | **NR** |
| **Heftdal 2021** | **269 patients** | **NR** | **NR** | **NR** | **NR** | **NR** |
| **Hensley 2022** | **984 patients** | **Not detailed** | **Not detailed** | **Not detailed** | **Not detailed** | **NR** |
| **Bociaga-Jasik 2023** | **121 patients** | **Not detailed** | **Headache: 38/121 (31.4%). Fever: 24/121 (19.83%).** | **0/121 (0%)** | **NR** | **NR** |
| **Oyaert 2022** | **27 patients** | **NR** | **NR** | **NR** | **NR** | **NR** |
| **Jongkees 2023** | **66 patients** | **NR** | **NR** | **NR** | **NR** | |

| | | | | | | |
|---|---|---|---|---|---|---|
| **Lamacchia 2022** | **8 PLWH** | **NR** | **NR** | **NR** | **NR** | **NR** |
| **Levy 2021** | **143 patients** | **Pain at injection site (39% after dose 1; 23.9% after dose 2).** | **Systemic AEs: 19.5% after dose 1; 47.9% after dose 2. Most common: fatigue, headache (dose 1); fatigue, fever (dose 2, usually <38°C).** | **NR** | **NR** | **NR** |
| **Lombardi 2022** | **71 PLWH** | **NR** | **NR** | **NR** | **NR** | **NR** |
| **Lv 2021** | **24 patients** | **NR** | **NR** | **NR** | **NR** | **NR** |
| **Madhi 2022** | **122 patients** | **Not detailed** | **Not detailed** | **Not detailed** | **Not detailed** | **NR** |

| | | | | | | |
|---|---|---|---|---|---|---|
| **Mauas 2023** | **595** | **Pain 145/595 (24.4%), Swelling 33/595 (5.6%), Redness 16/595 (2.7%), Induration 12/595 (2.0%)** | **Fatigue 142/595 (23.9%), Myalgias/Arthralgias 131/595 (22.0%), Headache 116/595 (19.5%), Fever 89/595 (15.0%), Abdominal pain 16/595 (2.7%), Diarrhea 16/595 (2.7%), Vomiting 7/595 (1.2%)** | **NR** | **NR** | **NR** |
| **Milano 2022** | **440 patients** | **Local pain: 170/440, (38.6%)** | **Fatigue: 94/440 (21.4%) Myalgia: 45/440 (10.2%) Fever: 37/440 (8.4%) Headache: 7/440 (1.6%) Lymphadenopathy: 2/440 (0.5%) Rash: 2/440 (0.5%)** | **1/697 (0.14%) – DVT** | **NR** | **NR** |
| **Nault 2022** | **106 patients** | **NR** | **NR** | **NR** | **NR** | **NR** |
| **Netto 2022** | **215 patients** | **Not detailed** | **Not detailed** | **Not detailed** | **Not detailed** | **NR** |

| | | | | | | |
|---|---|---|---|---|---|---|
| **Noe 2022** | **665 patients** | **NR** | **NR** | **NR** | **NR** | **NR** |
| **Sisteré-Oró 2022** | **10 patients** | **NR** | **NR** | **NR** | **NR** | **NR** |
| **Portillo 2022** | **131 PLWH** | **NR** | **Not detailed** | **Not detailed** | **None reported** | **NR** |
| **Pourcher 2022** | **97 PWH** | **NR** | **NR** | **NR** | **NR** | **NR** |
| **Rahav 2021** | **156 patients** | **40.6% after first dose (most common: pain at injection site)** | **NR** | **NR** | **NR** | **NR** |
| **Ruddy 2021** | **14 PWH** | **Mild – moderate pain: 13/14 (93%)** | **NR** | **NR** | **NR** | **NR** |
| **Schmidt 2022** | **50 HIV** | **NR** | **NR** | **NR** | **NR** | **NR** |
| **Speich 2022** | **341 patients** | **NR** | **NR** | **NR** | **NR** | **NR** |
| **Spinelli 2022** | **BNT162b2 group** | **NR** | **NR** | **NR** | **NR** | **NR** |

| | | | | | | |
|---|---|---|---|---|---|---|
| | **mRNA-1273 group** | **NR** | **NR** | **NR** | **NR** | **NR** |
| **Tortellini 2022** | **37 patients** | **NR** | **NR** | **NR** | **NR** | **NR** |
| **Tuan 2021** | **78 patients** | **NR** | **NR** | **NR** | **NR** | **NR** |
| **Verburgh 2023** | **195 PWH** | **NR** | **NR** | **NR** | **NR** | **NR** |
| **Vergori 2022** | **PCDR (<200): 44 patients** | **Not detailed** | **Not detailed** | **NR** | **NR** | **NR** |
| | **ICDR (200-500): 96 patients** | **Not detailed** | **Not detailed** | **Not detailed** | **Not detailed** | **Not detailed** |
| | **HCDR (>500): 76 patients** | **Not detailed** | **Not detailed** | **Not detailed** | **Not detailed** | **Not detailed** |
| **Wong 2022** | **BNT162b2: 423 patients** | **NR** | **NR** | **NR** | **NR** | **NR** |

| | | | | | | |
|---|---|---|---|---|---|---|
| | **CoronaVac / Sinovac: 184 patients** | **NR** | **NR** | **NR** | **NR** | **NR** |
| **Wu 2021** | **259 patients** | **Not detailed** | **Fatigue 0.8% (2/259), drowsiness 1.7% (4/259), fever 0.0% (0/259)** | **None** | **None** | **None** |
| **Wu 2023** | **138 patients** | **NR** | **NR** | **NR** | **NR** | **NR** |
| **Zeng 2022** | **132 patients** | **NR** | **NR** | **NR** | **NR** | **NR** |
| **Zou 2022** | **46 patients** | **NR** | **NR** | **NR** | **NR** | **NR** |
| **Aledo 2022** | **100 patients** | **NR** | **NR** | **0/100 (0%)** | **NR** | **NR** |
| **Chittrakarn 2024** | **CD4 ≤ 200 cells/µL: 15 patients** | **NR** | **NR** | **NR** | **NR** | **NR** |

| | | | | | | |
|---|---|---|---|---|---|---|
| | CD4 > 200 cells/µL: 15 patients | NR | NR | NR | NR | NR |
| Li 2023 | HIV-infected (Nadir ≤350): 59 patients | Pain at injection site: 9/108 (8.3%)<br>Swelling/induration/itch : 0/108 (0%) | Fatigue: 12/108 (11.1%)<br>Fever: 8/108 (7.4%)<br>Muscle/joint pain: 4/108 (3.7%)<br>Sore throat: 1/108 (0.9%) | None | None | None |
| | HIV-infected (Nadir >350): 42 patients | | | | | |
| Loubet 2022 | 897 patients | NR | NR | NR | NR | NR |
| Lv 2023 | 34 patients | Pain at the injection point: 2/34 (5.88%)<br>Muscle pain, joint pain: 1/34 (2.94%) | Fatigue: 2/34 (5.88%) | NR | NR | NR |
| Lu 2023 | 100 patients | Pain: 14/100 (14%)<br>Itch: 1/100 (1%) | Fatigue: 4/100 (4%)<br>Rash: 1/100 (1%) | NR | NR | NR |
| Morrocchi 2022 | 28 patients | NR | NR | 0/28 (0%) | None reported | NR |
| Wang 2024 | 228 patients | NR | NR | NR | NR | NR |

| | | | | | | |
|---|---|---|---|---|---|---|
| | | **NR** | **NR** | **NR** | **NR** | **NR** |
| **Yu 2023** | **236 patients** | **NR** | **NR** | **NR** | **NR** | **NR** |
